# Fruit–Carrot-Based Smoothies as Innovative Products with a Complex Matrix of Bioactive Compounds Effected on Activities of Selected Digestive Enzymes and Cholinesterases In Vitro

**DOI:** 10.3390/antiox12040917

**Published:** 2023-04-12

**Authors:** Emel Hasan Yusuf, Aneta Wojdyło, Ana Isabel Bourbon, Paulina Nowicka

**Affiliations:** 1 Department of Fruit, Vegetable and Plant Nutraceutical Technology, The Wrocław University of Environmental and Life Sciences, 37 Chełmońskiego Street, 51-630 Wrocław, Poland; 2 International Iberian Nanotechnology Laboratory, Department of Life Sciences, Av. Mestre José Veiga s/n, 4715-330 Braga, Portugal

**Keywords:** polyphenolic compounds, carotenoids, pro-health properties, sensory evaluation, fruit

## Abstract

In this study, four different carrot varieties (purple, yellow, white, and orange) were used in the production of smoothies with raspberry, apple, pear, strawberry, and sour cherry juices. The in vitro inhibition effects against α- amylase, α- glucosidase, pancreatic lipase, acetylcholinesterase, and butyrylcholinesterase were measured, bioactive compounds, physicochemical characteristics, including sensorial features were described. The antioxidant activities of the studied samples were analyzed using the ORAC, ABTS, and FRAP methods. The raspberry–purple carrot smoothie showed the highest antioxidant activity against lipase and butyrylcholinesterase enzyme activity. The sour cherry–purple carrot smoothie showed the highest total soluble solids, total phenolic acid, total anthocyanins, and procyanidin contents; dry mass; and osmolality. Although the apple–white carrot smoothie achieved the highest acceptance after sensorial evaluation, it did not exhibit any potent biological activities. Thus, food products with purple carrot, raspberry, and sour cherry ingredients are suggested as functional and/or novel matrix compositions with high antioxidant potential.

## 1. Introduction

In this era of high-speed technology and fast-food preferences, people do not have enough time to download an application on their PC and/or wait for the food or beverages ordered. Furthermore, the amount of fruit or vegetables consumed is insufficient to acquire essential vitamins and minerals that boost the immune system against viruses and/or microorganisms.

To boost the immune system and healthy body functions, fruit–vegetable juices, nectars, or smoothies, which can be easily consumed, are preferred instead of raw fruit and vegetables. A wide range of beverages with different ingredients is available in the market. Many of them have been investigated as functional beverages, such as sea buckthorn [1], pumpkin, and purple carrot (PC) blended smoothies [2]. In a research study, a carrot-blended tomato smoothie was applied with pumpkin, lemon juice, mineral water, and marine salt [3], which was found to increase the lycopene and carotene contents, with increased bioavailability. Thus, carrot-based smoothies can be a good choice for healthy beverage alternatives.

Carrot is a popular vegetable with high nutritional contents such as carotenoids, which are vitamin A precursors and essential for cell regulation and eyesight [4]; polyphenolics—phenolic acids and flavan-3-ols (orange, white, yellow, and purple carrots); and in the case of purple carrot, also anthocyanins, which are effective against aging, diabetes, cardiovascular diseases, cancers, and neurological disorders [5]. Moreover, the high vitamin and mineral contents of carrot might help reduce food-related deficiencies and are responsible for the proper conduct of biochemical reactions and functioning of the human body. Carrots are also a good source of dietary fiber (both soluble and insoluble fraction), which influences the inhibition of the absorption of dietary fats and their collection in the tissues of the liver. In addition, it influences lower blood glucose levels and improves peristalsis. 

Nevertheless, children may not desire to consume healthy carrot beverages for several reasons, such as taste, smell, and/or appearance. As role models, parents should support their children in consuming more healthy beverages. Fruit and vegetable snacks are not only healthy but also have additional benefits as they do not contain gluten, which is harmful to people with celiac disease [6]. Hence, plant-based ingredients might be a good alternative for all age groups for snacking.

Therefore, the aim of this study was to use purple (PC), yellow (YC), orange (OC), and white carrot (WC) varieties to produce smoothies with raspberry (RJ), apple (AJ), pear (PJ), strawberry (SJ), and sour cherry (SCJ) juices (the most popular fruit in Poland from the *Rosaceae* family), and to investigate the resulting smoothies with respect to the physicochemical characteristics, sensorial characteristics, bioactive compounds, and in vitro pro-health properties. Thus, the formulated research objective allowed for complete verification of the research hypothesis which assumes that the carrot can be a functional base in the production of smoothies dedicated to children in the form of a snack or a second breakfast, and that the addition of fruits from the *Rosaceae* family to the carrot base can shape the sensory and health-promoting value of the finished products, making them more desirable by the indicated target group. Furthermore, the research presented in this manuscript allows for the identification of the optimal directions for the use and application of carrots in the diet, industry, and food service. Consequently, the presented results may in the future bring measurable health-related and economic benefits worldwide.

## 2. Materials and Methods

### 2.1. The Technology of Smoothie Preparation

The PC, OC, YC, and WC varieties were purchased from Fusion Gusto (Dabrowa, Poland). Raspberry (*Rubus idaeus*), apple (*Malus domestica*), pear (*Pyrus communis*), strawberry (*Fragaria ^x^ ananassa*), and sour cherry (*Prunus cerasus*) were purchased from retail markets in Poland. These fruits were chosen because (i) all of them belong to the *Rosaceae* family; (ii) they are very popular and liked across the world; (iii) they mask undesirable flavors and appearance well; and (iv) they are readily available; therefore, all recipes can be reproduced easily.

Fruit juices were produced freshly using a laboratory hydraulic press (SRSE, Warsaw, Poland) and stored in a freezer until the production of carrot-based smoothies.

**Figure 1:**
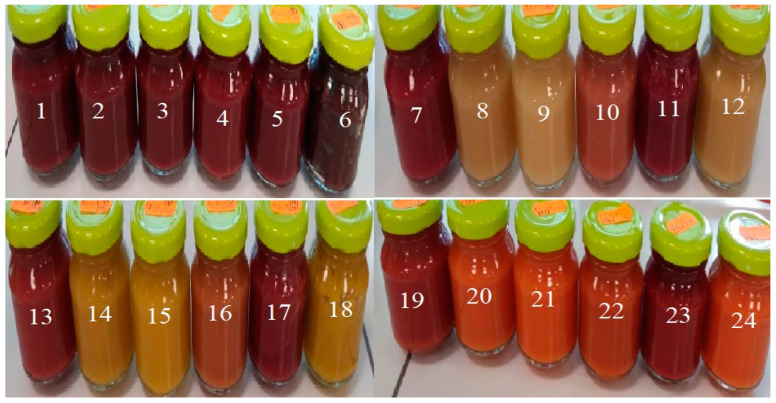
Prepared carrot blended smoothies (1, raspberry juice with purple carrot puree (RJ–PC); 2, apple juice with purple carrot puree (AJ–PC); 3, pear juice with purple carrot puree (PJ–PC); 4, strawberry juice with purple carrot puree (SJ–PC); 5, sour cherry juice with purple carrot puree (SCJ–PC); 6, purple carrot puree %100 (PC%100); 7, raspberry juice with white carrot puree (RJ–WC); 8, apple juice with white carrot puree (AJ–WC); 9, pear juice with white carrot puree (PJ–WC); 10, strawberry juice with white carrot puree (SJ–WC); 11, sour cherry juice with white carrot puree (SCJ–WC); 12, white carrot puree %100 (WC%100); 13, raspberry juice with yellow carrot puree (RJ–YC); 14, apple juice with yellow carrot puree (AJ–YC); 15, pear juice with yellow carrot puree (PJ–YC); 16, strawberry juice with yellow carrot puree (SJ–YC); 17, sour cherry juice with yellow carrot puree (SCJ–YC); 18, yellow carrot puree %100 (YC%100); 19, raspberry juice with orange carrot puree (RJ–OC); 20, apple juice with orange carrot puree (AJ–OC); 21, pear juice with orange carrot puree (PJ–OC); 22, strawberry juice with orange carrot puree (SJ–OC); 23, sour cherry juice with orange carrot puree (SCJ–OC); 24, orange carrot puree %100 (OC%100)).

Raw carrot materials were washed and sliced, and 1% L-ascorbic acid (10% *w*/*v*) was added to the carrot slices and disintegrated for 30 s in a Thermomix (Vorwerk, Wuppertal, Germany). The carrot purees thus prepared were pasteurized at 95 °C in the Thermomix for 1 min and then mixed with fruit juices in a 1:1 ratio. The mixtures were mixed at 90 °C for 30 s in the Thermomix and pasteurized at 95 °C for 1 min. The resulting smoothies were transferred to sterilized glass jars, self-pasteurized for 10 min, and cooled to room temperature (20 °C) until further analysis. The 20 carrot-blended smoothies obtained and the four carrot purees (control groups) are shown in Figure 1.

### 2.2. Physicochemical Characteristics

The L-ascorbic acid content of the samples was determined according to the PN-A-04019. The TSS content was measured using a portable refractometer (Atago RX-5000, Atago Co. Ltd., Saitama, Japan) and expressed in “°Brix.” The dry mass was evaluated as follows: the samples were mixed with diatomaceous earth, pre-dried, and then subjected to final drying under reduced pressure. The TA was determined by titration against 0.1 N NaOH to an endpoint of pH 8.1 using an automatic pH titration system (pH meter type IQ 150; Warsaw, Poland), and the pH was measured using the same equipment. The TA and dry mass were determined according to the PN norms PN-EN 12145:2001 and PN-EN12145:2000, respectively. The viscosity was measured using a rotation viscometer MC1 (DV-II+ PRO VISCOMETER, Brookfield, England) at 20 °C and expressed as mPas. The pectin content was determined according to Pijanowski et al. [7]. The osmotic strength was evaluated using an osmometer (Marcel OS 3000). The ash (%), sugar, and organic acid contents (g/100 mL) were evaluated according to Wojdyło et al. [8]. The ash content was measured using the furnace method, individual sugars were determined using HPLC-ELSD (High-Performance Liquid Chromatography-Evaporative Light Scattering Detector), and organic acids were determined using UPLC-PDA (Ultra-Performance Liquid Chromatography-Photodiode Array Detection). The mineral content was determined using an atomic absorption spectrophotometer (AA-7000F/AAC SHIMADZU, Shimadzu Corporation). Color parameters (CIEL*a*b*) of the samples were quantified using a Color Quest XE Hunter Lab colorimeter (Reston, VA, USA). The L* (lightness), a* (redness–greenness), and b* (yellowness–blueness) values were determined using the CIE standard Illuminant D65 at a 10° observer angle. All measurements were performed in triplicate.

### 2.3. Identification and Quantification of Polyphenolics and Carotenoids

Phenolic compounds were identified using LC/MS-Q-Tof (Liquid Chromatography/Mass Spectrometry-Quadrupole Time-of-flight) (Waters, Manchester, UK). The observed retention times and spectra were compared with the standards. The polyphenolic content was quantified using external calibration curves compared with the standards and investigated using LC/MS and UPLC, according to Wojdyło et al. [8]. About 2 g of the carrot-blended smoothies was mixed with 8 mL of HPLC-grade methanol–H_2_O mixture (30:70%, *v*/*v*), ascorbic acid (2%), and acetic acid (1%), and was then sonicated (Sonic 6D, Polsonic, Warsaw, Poland) for 15 min. Then, the samples were stored in a refrigerator for 24 h and centrifuged at 19,000× *g* for 10 min. The supernatant was filtered through a 0.20-μm hydrophilic polytetrafluoroethylene (PTFE) membrane (Millex Simplicity Filter, Merck, Darmstadt, Germany) and used for further analysis. All samples were measured in triplicate and expressed as mg per 100 mL.

The carrot-based smoothies, which contained 10% MgCO_3_ and 1% butylhydroxytoluene, were shaken with 5 mL of a ternary mixture of methanol, acetone, and hexane (1:1:2, by vol.) at 300 rpm for 30 min in the dark to prevent oxidation. The supernatants were acquired after four re-extractions from solid residues. The combined fractions were obtained after centrifugation (4 °C, 7 min at 19,000× *g*; MPW-350, Warsaw, Poland) and evaporated. The pellet was subtilized using 2 mL of 100% methanol, filtered through a 0.20-μm hydrophilic PTFE membrane, and used for further analysis. The carotenoid content was analyzed at 30 °C using LC-MS-Q/TOF (identification) and UPLC-PDA (quantification) on an ACQUITY UPLC BEH RP C18 column protected by a guard column of the same material. The elution solvents were a linear gradient of acetonitrile–methanol mixture (70:30%, *v*/*v*) (A) and 0.1% formic acid (B) at a flow rate of 0.42 mL/min. This procedure was monitored at 450 nm. All measurements were performed in triplicate and expressed as mg per 100 mL.

### 2.4. Analyses of In Vitro Antioxidant and Biological Activities

For all antioxidant and biological assays, about 5 gr of the supernatant of carrot-based smoothies was used. The antioxidant activity of the carrot-blended smoothies was determined using ABTS, FRAP, and ORAC assays according to Re et al. [9], Benzie and Strain [10], and Ou et al. [11], respectively. It was measured by the reduction of ABTS+• radicals, and the absorbance was read at 734 nm. The ferric reduction of the samples was determined using the FRAP assay. At low pH, the colorless ferric complex (Fe3+-tripyridyltriazine) changed to a blue ferrous complex (Fe^2+^-tripyridyltriazine) by the action of electron-donating antioxidants, and the absorbance was read at 593 nm. The ORAC of the smoothies was determined using a spectrofluorometric method, and fluorescence decreased with the oxidation of free radicals in the presence of antioxidants.

Antidiabetic and antiobesity activities of the carrot-blended smoothies were investigated to understand α-amylase, α-glucosidase [12], and lipase inhibitory effects [13]. The α-amylase enzyme inhibition activity was analyzed using the reaction of iodine with starch after incubation at 37 °C, and the absorbance was read at 600 nm. The α-glucosidase enzyme inhibition activity was evaluated using the reaction of the enzyme with the β-D-glucosidase substrate, and the absorbance was read at 405 nm. The pancreatic lipase enzyme inhibition activity was analyzed using *p*-nitrophenol formed from *p*-nitrophenyl acetate after incubation at 37 °C, and the absorbance was read at 400 nm. The antiaging activity was determined using AChE and BuChE, according to Gironés-Vilaplana et al. [14]. The reaction of thiocholine with 5,5′-dithiobis-(2-nitrobenzoic acid) produced 2-nitrobenzoate-5-mercaptothiocholine and 5-thio-2-nitrobenzoate. The absorbance of the substrates of acetylcholine iodine and butylcholine chloride was read at 405 nm.

All tests to evaluate the antioxidant activity (ABTS, FRAP, and ORAC); α-amylase, α-glucosidase, and lipase inhibition effects; and anticholinergic activity were performed in triplicate using a microplate reader (Synergy TM H1; BioTek, Winooski, VT, USA).

### 2.5. Sensorial Evaluation

Sensory tests were carried out in a sensory analysis laboratory equipped with individual booths (at a controlled temperature of ~20 °C under combined natural/artificial light) designed according to the ISO 8589:2009 standards. The sensory laboratory was located at the Faculty of Biotechnology and Food Sciences of the Wrocław University of Environmental and Life Sciences (Poland). Nine fully trained panelists aged 25–43 years conducted the sensory evaluation sessions from 10 a.m. to 1 p.m. All panelists were provided with the same training to make them accustomed to the sensory attributes of smoothies and to understand the descriptors being used because they had to be able to identify differences between products, describe different product attributes (qualitatively), and scale the intensity of the attributes (quantitatively). In addition, all panelists were nonsmokers.

Sensory evaluation of the carrot-based smoothies was carried out using a 9-point hedonic scale (like extremely, like very much, like moderately, like slightly, neither like nor dislike, dislike lightly, dislike moderately, dislike very much, and dislike extremely). The panelists voted for the smoothies based on their appearance, sweetness, carrot taste, which fruit they sense most (raspberry, apple, pear, strawberry, and sour cherry), carrot smell, and delight. The smoothies were assigned codes for sensory evaluation and evaluated at room temperature. The smoothie samples were served in small white plastic glasses. After each sample, the panelists drank water to neutralize the taste in their mouths for the next sample.

According to the national laws, no ethical approval was required for this study. The panelists were informed about the study’s aim and that their participation was entirely voluntary so they could stop the evaluation at any point and the responses would be anonymous.

### 2.6. Statistical Analyses

Results were subjected to analysis of variance (*p* < 0.05), and Tukey’s honestly significant difference tests (Tukey’s multiple comparisons of means 95% familywise confidence level) were performed using the R software (version 4.1.2, R Core Team, Austria). The results were demonstrated as mean values (*n* = 3) ± standard deviation.

## 3. Results and Discussion

### 3.1. Physicochemical Characteristics of the Carrot-Blended Smoothies

The L-ascorbic acid, pectin, TSS, and ash contents; viscosity; pH; TA; dry mass; osmolality; and color characteristics of the carrot-blended smoothies are presented in Table 1, with significant differences (*p* ≤ 0.05).

The L-ascorbic acid content is essential for normal body functions such as the synthesis of folic acid, tyrosine, and tryptophan; hydroxylation of proline, glycine, carnitine, lysine, and catecholamine; and iron absorption, besides acting as an antioxidant agent [15]. Moreover, although fruit and vegetables are rich in vitamin C, the amount of vitamin C decreases during food processing due to heating and air exposure [16]. In the present study, L-ascorbic acid was added to the smoothies to prevent enzyme activity and color changes. The L-ascorbic acid content in the smoothies ranged from 76.98 mg/100 mL to 196.76 mg/100 mL. The highest L-ascorbic acid content was observed in the AJ–YC (196.76 mg/100 mL), PJ–WC (173.65 mg/100 mL), and AJ–OC (171.27 mg/100 mL) smoothies, whereas the lowest L-ascorbic acid content was observed in the SCJ–OC smoothie (89.13 mg/100 mL). Thus, AJ provides the highest amount of vitamin C to YC and OC purees.

Viscosity is one of the characteristics of liquid food products [17]. In the present study, the viscosities of the carrot-blended smoothies ranged from 3.30 to 54.08 mPas. The viscosities of the control groups of PC100% and YC100% were more than 100 mPas; in the case of WC, viscosity was 49 mPas, whereas in OC puree, it was 15 mPas. Nevertheless, the highest viscosity was observed in the PJ–PC (54.08 mPas) and SJ–YC (49.05 mPas) smoothies, whereas the lowest viscosity was observed in the AJ–OC smoothie (3.30 mPas). This results directly from the characteristics of the individual purees. The addition of those that were characterized by the highest viscosity (PC and YC puree) resulted in the fact that products based on them were more dense than those with the addition of OC puree. In the case of smoothie development, viscosity is an extremely important factor that shapes the quality of the final product. By design, this product must be semifluid and drinkable. Therefore, when designing a smoothie based on PC or YC, it is worth considering using a larger proportion of juice than puree, so that the final viscosity does not exceed 45 mPa (intrinsic viscosity in the opinion of the sensory panel).

In a previous study, TA and pH were evaluated together to determine food quality [18]. In the present study, TA ranged from 0.15 to 1.21 g/100 mL. The highest TA was observed in the RJ–PC (1.21 g/100 mL), RJ–OC (1.17 g/100 mL), and RJ–YC (1.17 g/100 mL) smoothies, whereas the lowest TA was observed in the PJ–OC (0.24 g/100 mL) and PJ–YC (0.25 g/100 mL) smoothies, except for the control groups, which included only carrot purees. Hence, RJ smoothies showed the highest TA, whereas PJ smoothies showed the lowest TA. Regarding pH values, the control groups showed the highest pH values in the following order: YC > OC > PC > WC. Besides the control groups, the highest pH values were observed in the PJ–PC (4.77), PJ–OC (4.70), and PJ–YC (4.69) smoothies, whereas the lowest pH values were observed in the RJ–WC (3.62) and RJ–OC (3.67) smoothies. These results demonstrated that carrot purees show high pH, but RJ shows an acidic pH. High acidic conditions prevent microbial activities and provide beverage stability [19]. Hence, TA and pH showed a contrasting finding that RJ–PC showed the highest TA value but with a low pH value.

Pectin plays the role of an immunomodulator in allergies [20] and protects against cardiovascular diseases [21]. Moreover, soluble dietary fibers (e.g., pectins) play a vital role in increasing gastrointestinal activities [22] and decreasing serum cholesterol [23]. Insoluble dietary fibers such as pectic polysaccharides, hemicellulose, and cellulose of carrot pomace also provide important health benefits such as reducing lipid and cholesterol levels [24]. In the present study, the highest pectin content was observed in the following order: PC100% > RJ–YC (1.10%) > WC100% > SCJ–PC (0.96%) > SCJ–OC (0.94%). However, the lowest pectin content was observed in the AJ–OC (0.26%) and SJ–OC (0.27%) smoothies. Based on these results, the SCJ–PC smoothie showed a higher pectin content than the SCJ–OC smoothie.

The TSS content influences the sweetness of food products due to the presence of soluble proteins and organic materials [25]. In the present study, the TSS content of carrot-blended smoothies ranged from 7.40 to 12.40 ˚Brix. The highest TSS content was observed in the SCJ–PC (12.40 ˚Brix) and AJ–PC (12.20 ˚Brix) smoothies, whereas the lowest TSS content was observed in the SJ–OC (7.40 ˚Brix), SJ–WC (7.60 ˚Brix), and SJ–YC (7.80 ˚Brix) smoothies. Therefore, the PC puree provided a higher sugar content, as shown by the high TSS content, but SJ reduced the sugar content and TSS.

The dry mass of the carrot-blended smoothies showed the same trend as the TSS content. The highest dry mass was observed in the SCJ–PC (13.11%) and AJ–PC (12.80%) smoothies, whereas the lowest dry mass was observed in the SJ–OC (8.27%), SJ–YC (8.53%), and SJ–WC (8.71%) smoothies. Nevertheless, the ash content of the carrot-based smoothies showed a different trend from that of the TSS and dry mass.

Osmolality determines the bioavailability of beverages in body hydration [26]. In the present study, the highest osmolality was observed in the SCJ–PC (804 mOsm/L), SCJ–YC (750 mOsm/L), and SCJ–WC (690 mOsm/L) smoothies, whereas the lowest osmolality was observed in the SJ–OC (419 mOsm/L), SJ–PC (444 mOsm/L), and SJ–YC (446 mOsm/L) smoothies. Hence, SCJ smoothies showed the highest osmolality, which shows that SCJ–carrot smoothies are not only rich in bioactive contents but are also easily absorbed by the digestive system. However, SJ–carrot smoothies showed the lowest osmolality.

The color of food products is an important parameter that increases consumer purchase rates. In the present study, the mean lightness (L*) of the carrot-based smoothies ranged from 32.21 to 55.58. The highest lightness was observed in PJ and AJ with WC and YC purees, whereas the lowest lightness (i.e., with the darkest color) was observed in the SCJ–WC (31.79), RJ–PC (32.21), and SCJ–YC (32.55) smoothies. Thus, as expected, SCJ and RJ provided the darkest colors, whereas AJ and PJ provided the lightest colors.

Moreover, other parameters such as a* (redness–greenness) and b* (yellowness–blueness) were also measured in the present study. The highest redness (the highest a* results) was observed in the OC puree and RJ, AJ, and PJ smoothies, whereas the highest greenness was observed in the WC puree and PJ and AJ smoothies. The highest yellowness was observed in the OC puree and AJ and PJ smoothies, whereas the highest blueness was observed in the SCJ–WC (6.50), SCJ–PC (7.98), and RJ–WC (8.21) smoothies. Hence, similar to lightness results, SCJ and RJ provided the dark blue color.

**Figure 2:**
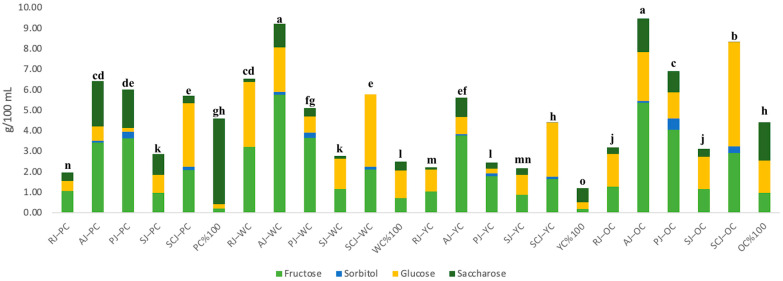
The total sugars (g/100 mL) in carrot-blended smoothies, letters above columns indicate statistical significance at *p* ≤ 0.05 according to Tukey’s test (the same letter = not significantly different). Total sugar content: RJ–PC, 1.97n. AJ–PC, 6.41cd. PJ–PC, 6.00de. SJ–PC, 2.87k. SCJ–PC, 5.72e. PC%100, 4.59gh. RJ–WC, 6.53cd. AJ–WC, 9.22a. PJ–WC, 5.11fg. SJ–WC, 2.78k. SCJ–WC, 5.77e. WC%100, 2.50l. RJ–YC, 2.22m. AJ–YC, 5.61ef. PJ–YC, 2.45l. SJ–YC, 2.16mn. SCJ–YC, 4.42h. YC%100, 1.21o. RJ–OC, 3.19j. AJ–OC, 9.47a. PJ–OC, 6.91c. SJ–OC, 3.11j. SCJ–OC, 8.35b. OC%100, 4.40h.

**Figure 3:**
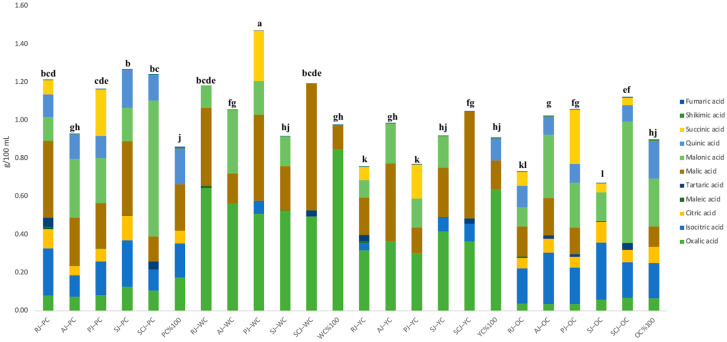
The organic acids (g/100 mL) in carrot-blended smoothies, letters above columns indicate statistical significance at *p* ≤ 0.05 according to Tukey’s test (the same letter = not significantly different). Total organic acid content: RJ–PC, 1.21bcd. AJ–PC, 0.93gh. PJ–PC, 1.17cde. SJ–PC, 1.27b. SCJ–PC, 1.24bc. PC%100, 0.86j. RJ–WC, 1.18bcde. AJ–WC, 1.06fg. PJ–WC, 1.47a. SJ–WC, 0.92hj. SCJ–WC, 1.19bcde. WC%100, 0.98gh. RJ–YC, 0.76k. AJ–YC, 0.99gh. PJ–YC, 0.77k. SJ–YC, 0.92hj. SCJ–YC, 1.05fg. YC%100, 0.91hj. RJ–OC, 0.73kl. AJ–OC, 1.02g. PJ–OC, 1.06fg. SJ–OC, 0.67 l. SCJ–OC, 1.12ef. OC%100, 0.90hj.

Sugar and organic acid contents of the carrot-blended smoothies are shown in Figure 2 and Figure 3, with significant differences (*p* ≤ 0.05).

In the present study, the presence of fructose, sorbitol, glucose, and saccharose was observed in carrot-based smoothies. Fructose levels ranged from 0.87 to 5.74 g/100 mL. The highest fructose content was observed in the AJ–WC (5.74 g/100 mL), AJ–OC (5.37 g/100 mL), and PJ–OC (4.04 g/100 mL) smoothies, whereas the lowest fructose content was observed in the SJ–YC (0.87 g/100 mL), SJ–PC (0.97 g/100 mL), and RJ–PC (1.07 g/100 mL) smoothies. Thus, the OC puree and AJ smoothies showed the highest fructose content. However, sorbitol was not determined in all samples. The highest sorbitol content was observed in the PJ–OC (0.55 g/100 mL), PJ–PC (0.33 g/100 mL), and SCJ–OC (0.32 g/100 mL) smoothies. The sorbitol content was not observed in the RJ–PC, SJ–PC, RJ–WC, SJ–WC, RJ–YC, SJ–YC, RJ–OC, and SJ–OC smoothies. Hence, the sorbitol content was high in the OC puree and PJ smoothies. The highest glucose content was observed in the SCJ–OC (5.07 g/100 mL), SCJ–WC (3.54 g/100 mL), and RJ–WC (3.18 g/100 mL) smoothies. The lowest glucose content was observed in the PJ–PC (0.16 g/100 mL), RJ–PC (0.47 g/100 mL), and AJ–PC (0.68 g/100 mL) smoothies. Therefore, the WC puree and SCJ samples were rich in glucose. The highest saccharose content was observed in the AJ–PC (2.22 g/100 mL), PJ–PC (1.87 g/100 mL), and AJ–OC (1.65 g/100 mL) smoothies, whereas saccharose was not observed in the SCJ–WC smoothie, and the lowest saccharose content was observed in the SCJ–YC (0.02 g/100 mL) and SCJ–OC (0.04 g/100 mL) smoothies. Thus, the PC puree and AJ samples were rich in saccharose. To summarize, the total sugar content was highest in the AJ–OC (9.47 g/100 mL), AJ–WC (9.22 g/100 mL), and SCJ–OC (8.35 g/100 mL) smoothies and lowest in the RJ–PC (1.97 g/100 mL), SJ–YC (2.16 g/100 mL), and RJ–YC (2.22 g/100 mL) smoothies. The OC puree and AJ samples showed the highest total sugar content.

Organic acids provide a specific smell and taste to fruit- and vegetable-based food [27]. In the present study, the presence of oxalic acid, isocitric acid, citric acid, maleic acid, tartaric acid, malic acid, malonic acid, quinic acid, succinic acid, shikimic acid, and fumaric acid was identified from the carrot-blended smoothies. Oxalic acid and fumaric acid were present in all smoothies, whereas other organic acids were observed in different amounts in each sample. The highest oxalic acid content was observed in the WC100% (0.85 g/100 mL), RJ–WC smoothie (0.64 g/100 mL), YC100% (0.64 g/100 mL), and AJ–WC smoothie (0.56 g/100 mL), and the highest fumaric acid content was observed in the PC100% (0.004 g/100 mL) and RJ–PC smoothie (0.003 g/100 mL). Thus, WC samples were rich in oxalic acid [19], and PC samples were rich in fumaric acid. The RJ–PC smoothie was the only sample that showed the presence of all organic acids studied. In summary, the PC puree samples were rich in total organic acids, whereas the OC puree samples showed a low total organic acid content.

Mineral contents of the carrot-blended smoothies are shown in Table 2, with significant differences (*p* ≤ 0.05).

Minerals are essential for homeostasis, and their deficiencies may cause diseases [28]. In the present study, sodium (Na), potassium (K), calcium (Ca), iron (Fe), and magnesium (Mg) were identified in carrot-based smoothies. The highest Na content was observed in all YC puree samples, whereas the lowest Na content was observed in the RJ–PC (20.65 mg/100 mL) and AJ–WC (21.70 mg/100 mL) smoothies. The highest K content was observed in the following order: SCJ–YC > RJ–YC > SCJ–PC, whereas the lowest K content was observed in the following order: SJ–OC < PJ–OC < AJ–WC. Thus, the YC puree and SCJ smoothies were rich in K. The highest Ca content was observed in the following order: RJ–YC > SCJ–PC > AJ–PC, whereas the lowest Ca content was observed in the following order: PJ–OC < AJ–OC < SCJ–OC. Therefore, PC puree samples were rich in Ca. The highest Fe content was observed in the following order: RJ–YC > AJ–PC > SCJ–PC > RJ–PC, whereas the lowest Fe content was observed in the following order: AJ–WC = SCJ–WC < SJ–WC. Thus, the PC puree and RJ smoothies were rich in Fe. Moreover, the highest Mg content was observed in the following order: SCJ–PC > RJ–YC > AJ–PC, whereas the lowest Mg content was observed in the following order: AJ–OC < SJ–WC. Thus, the PC puree samples were rich in Mg. According to the literature, mineral contents and ash contents are related to each other. In the present study, the highest ash content was observed in the SCJ–YC (0.84%), SJ–YC (0.74%), and SCJ–PC (0.72%) smoothies, whereas the lowest ash content was observed in the PJ–OC (0.27%), AJ–OC (0.29%), and SJ–OC (0.30%) smoothies. Thus, YC puree samples showed the highest ash content, whereas OC puree samples showed a lower ash content.

### 3.2. Identification and Quantification of Polyphenolics and Carotenoids

Raw carrot materials do not contain flavanols and flavan-3-ols [29]; however, due to the addition of fruit juices in the present study, the carrot-blended smoothies were rich in flavan-3-ols, phenolic acids, flavanols, anthocyanins, and polymeric procyanidins. The phenolic content of the carrot-based smoothies is shown in Table 3 (qualitatively), and Supplementary S1 (quantitatively) with significant differences (*p* ≤ 0.05).

Flavan-3-ols exhibit activities against oxidation, carcinogens, microbes, and neurological diseases [30]. In the present study, procyanidin B2 ([M–H]− at m/z = 577), procyanidin B4 ([M–H]− at m/z = 577), and epicatechin ([M–H]− at m/z = 289) were identified, and flavan-3-ols were quantified in carrot-blended smoothies. Procyanidin B2 was identified in only RJ, AJ, SJ, and SCJ blended with the PC puree, procyanidin B4 in RJ, PJ, and SJ blended with carrot. Therefore, flavan-3-ols were not observed in raw carrot materials [29]; however, after processing and the addition of different fruit juices, the flavan-3-ol content increased and ranged from 12.14 to 127.93 mg/100 mL in the smoothies. The highest total flavan-3-ol content was observed in the SCJ–OC (127.93 mg/100 mL), SCJ–YC (113.22 mg/100 mL), and RJ–PC (84.08 mg/100 mL) smoothies, whereas the lowest total flavan-3-ol content was observed in the PJ–OC (12.14 mg/100 mL), PJ–WC (19.89 mg/100 mL), and PJ–YC (25.07 mg/100 mL) smoothies. Thus, SCJ samples were rich in flavan-3-ols.

As reported in a previous study, phenolic acids show antioxidant and anti-inflammatory activities [31]. In our previous work, fourteen phenolic acids were identified and quantified from raw carrot materials [29]. However, in the present study, 13 different phenolic acids were observed in the carrot-blended smoothies. In all samples, 5-o-caffeoylquinic acid ([M–H]− at m/z = 353), 4-o-feruloylquinic acid ([M–H]− at m/z = 367), and di-ferulic acid derivatives ([M–H]− at m/z = 527) were identified. The highest phenolic acid content was observed in all samples after the addition of SCJ, followed by PJ. In the smoothies, cis-5-*p*-coumaroylquinic acid ([M–H]− at m/z = 337) and *p*-coumaric acid ([M–H]− at m/z = 325) were newly identified phenolic acids. The cis-5-*p*-coumaroylquinic acid and *p*-coumaric acid were only identified in the SCJ–PC smoothie. Moreover, PC puree smoothies showed the highest number of different types of phenolic acids and total phenolic acid content. The lowest phenolic acid content was observed in the following order: RJ–WC < RJ–YC < SJ–WC. Hence, the PC puree and SCJ samples showed the highest phenolic acid content, whereas the WC puree and RJ samples showed the lowest phenolic acid content.

Flavanols promote blood flow to the brain and heart, decrease blood pressure, and prevent cell damage [32]. In the present study, only quercetin-3-galactoside ([M–H]− at m/z = 609) and genistin ([M–H]− at m/z = 269) were identified and quantified from the carrot-blended smoothies. Moreover, genistin resulted from SCJ, and quercetin-3-galactoside resulted from RJ, SJ, and SCJ. The highest total flavanol content was observed in the following order: SCJ–WC > SCJ–YC > SCJ–OC. Thus, sour cherry was rich in flavanols.

Anthocyanins protect against type 2 diabetes, cardiovascular diseases, and cancer [33]. In the present study, seven anthocyanins were quantified; however, from raw PC, only five different anthocyanins were quantified [29]. In the present study, RJ, SJ, and SCJ increased the anthocyanin content of carrot-blended smoothies. Cyanidin-3-o-xylosyl-galactoside ([M–H]− at m/z = 581) and cyanidin-3-o-glucosyl-rutinoside resulted from RJ and SCJ, whereas cyanidin-3-arabinoside resulted only from RJ; cyanidin-3-o-xylosyl-*p*-coumaroylglucosyl-galactoside resulted from SJ, and cyanidin-3-o-xylosyl-cinpoyl-glucosylgalactoside resulted from SCJ. The highest total anthocyanin content was observed in the following order: SCJ–PC > SCJ–OC > SCJ–WC. Therefore, similar to the flavanol content, sour cherry was also rich in anthocyanins

Polymeric procyanidins exhibit anticancer, anti-inflammatory, antioxidant, and antiallergenic characteristics [34]. In the present study, the procyanidin content ranged from 4.26 to 25.56 mg/100 mL in the smoothies. The highest polymeric procyanidin content was observed in the following order: SCJ–PC > SCJ–YC > SCJ–WC, whereas the lowest polymeric content was observed as follows: PJ–PC < PJ–WC < PJ–YC. Thus, SCJ increased the polymeric procyanidin content of the carrot-based smoothies, whereas PJ decreased it. Moreover, in the present study, the DP (the number of flavanol units) of polymeric procyanidins was explored. The highest DP values were observed in the following order: RJ–PC > AJ–PC > PJ–PC. However, DP was not observed in the SJ–PC, SJ–WC, SJ–YC, and SJ–OC smoothies. Hence, these results show that PC changes the DP values, but SJ demonstrates the antagonistic characteristic with carrot, and the DP was not determined.

Carrot is a popular vegetable with a high carotenoid content; carotenoids are essential bioactive chemicals, vitamin A precursors, and anticancer, antidiabetic, antibacterial, and neuroprotective agents [35]. The carotenoid content of the carrot-based smoothies is shown in Table 3 and Supplementary S1, with significant differences (*p* ≤ 0.05).

In the present study, only four carotenoids were identified: α-cryptoxanthin (zeinoxanthin), β-carotene, pheophytin a, and lutein. However, raw carrots showed 12 carotenoid types [29]. Thus, the smoothie manufacturing processes followed decreased the carotenoid types and contents. The primary reasons might be heating, air, light, and water exposures of carotenoids [36].

As PC is rich in α-cryptoxanthin, α-cryptoxanthin and β-carotene were quantified only in the AJ–PC smoothie [29]. However, PC did not contain β-carotene [29], and it resulted from AJ. Therefore, the highest total carotenoid content was observed in the following order: AJ–PC > SJ–PC > PJ–PC, but carotenoids were absent in the RJ–PC smoothie and all WC-based smoothies. 

### 3.3. Analyses of In Vitro Antioxidant and Biological Activities 

The antioxidant activities (ABTS, FRAP, and ORAC) of the carrot-blended smoothies are shown in Table 4, with significant differences (*p* ≤ 0.05).

Bioactive compounds in fruit- and vegetable-based food products increase their antioxidant characteristics [37]. In the present study, the ABTS antioxidant activity ranged from 0.42 to 1.78 mmol Trolox/100 mL. The highest activity was observed in PC puree samples in the following order: RJ > SJ > SCJ. However, the lowest ABTS activity was observed in PJ samples. Thus, these results showed that dark-colored fruit and vegetables increase the antioxidant activities of food and beverages. Similar results were observed for the FRAP and ABTS activities. However, unlike the ABTS activity, the SCJ–YC smoothie showed the lowest FRAP activity. Moreover, the ORAC activity ranged from 0.03 to 0.42 mmol Trolox/100 mL. The highest ORAC activity was observed in the following order: RJ–PC > SCJ–OC > SCJ–PC, whereas the lowest ORAC activity was observed in PJ smoothies. Thus, PC, RJ, and SCJ increased the antioxidant characteristics of smoothies. These samples were rich in flavon-3-ols, phenolic acids, flavanols, anthocyanins, and procyanidins. It should be emphasized that in the case of the developed products, not the quantity, but the quality of bioactive compounds had a greater impact on the shape of antioxidant potential of the final products. This was also indicated by other authors [1; 8; 12], who showed the high antioxidant activity of polymerized compounds and anthocyanins, which was confirmed in this study. The potential of polyphenolic compounds has been known for a long time, and the conducted research confirms this fact, indicating that the fortification of carrot puree with fruit juices, which are donors of secondary metabolites of plants, allows for the shaping of the health-promoting properties in the final products.

The results of the in vitro biological activities of carrot-blended smoothies are shown in Table 4, with significant differences (*p* ≤ 0.05) for α-amylase [IC50], α-glucosidase [IC50], lipase [IC50], AChE [% inhibition], and BuChE [% inhibition].

As reported in a previous study, the inhibition of α-amylase and α-glucosidase may control diabetes [38]. In the present study, the highest α-amylase inhibition activity was observed in the following order: SJ–PC > SCJ–YC > AJ–PC. However, the lowest α-amylase inhibition activity was observed as follows: PJ–WC < SCJ–WC < AJ–OC < SJ–YC. Thus, PC samples showed the highest α-amylase inhibition activity. Nevertheless, the highest α-glucosidase inhibition activity was observed in the following order: RJ–OC > SJ–WC > SCJ–YC, whereas the lowest α-glucosidase inhibition activity was observed as follows: AJ–PC < PJ–YC < PJ–PC. SJ, SCJ, and AJ smoothies showed the highest α-amylase inhibition activity, and RJ, SJ, and SCJ smoothies showed the highest α-glucosidase inhibition activity. The PC puree showed the lowest α-glucosidase inhibition activity, whereas the YC puree showed the lowest α-glucosidase and α-amylase inhibition activities. Hence, α-amylase inhibition activity might be related to the content of anthocyanins, but also to the content of pectins, whereas α-glucosidase inhibition activity is due to the interaction with polymeric procyanidins, flavanol, and also organic acids.

The inhibition of pancreatic lipase might decrease obesity [39]. In the present study, the highest lipase inhibition activity was observed in the following order: RJ–PC > SCJ–PC > RJ–WC, whereas the lowest lipase inhibition activity was observed as follows: PJ–OC < PJ–YC < PJ–WC. Therefore, the RJ–PC smoothie showed the highest lipase inhibition activity. Moreover, similar to α-amylase inhibition activities, anthocyanins and pectins might be responsible for lipase inhibition activities.

The inhibition of AChE and BuChE might reduce nervous system disorders related to aging [40]. In the present study, the highest AChE inhibition activity was observed in the following order: SCJ–YC > RJ–PC > RJ–YC, whereas the lowest AChE inhibition activity was observed as follows: PJ–OC < AJ–YC < SJ–OC. Thus, RJ and SCJ smoothies showed an increased AChE inhibition activity. Moreover, the highest BuChE inhibition activity was observed in the following order: RJ–PC > RJ–YC > SJ–PC, whereas the lowest BuChE inhibition activity was observed as follows: PJ–WC < AJ–YC < AJ–OC. Similar to the lipase inhibition activity, the RJ–PC smoothie showed the highest BuChE inhibition activity as well. Moreover, its lipase, AChE, and BuChE inhibition activities were similar to the results of the antioxidant activity assays, which identify it as the best product. Thus, the anthocyanin content increased the enzyme inhibition and antioxidant activities of the carrot-blended smoothies. Furthermore, the RJ–PC smoothie showed the highest polymeric procyanidin content, and these bioactive compounds might also shape the activities.

To sum up, the obtained products, in particular those developed on the basis of PC puree, show a pro-health effect at the level of in vitro research. On the one hand, processing processes (e.g., heating and oxidation) affect the degradation of polyphenolic compounds compared to fresh raw materials. On the other hand, creating recipes and combining several plant materials can cause a synergy effect, which can ultimately increase the health-promoting effect. However, in order to demonstrate the health-promoting effect with certainty in the future, selected formulations should be tested on a human model, taking into account physiological processes, digestive processes, and biotransformation that may change the activities in the body.

### 3.4. Sensorial Evaluation of Carrot-Blended Smoothies

**Figure 4:**
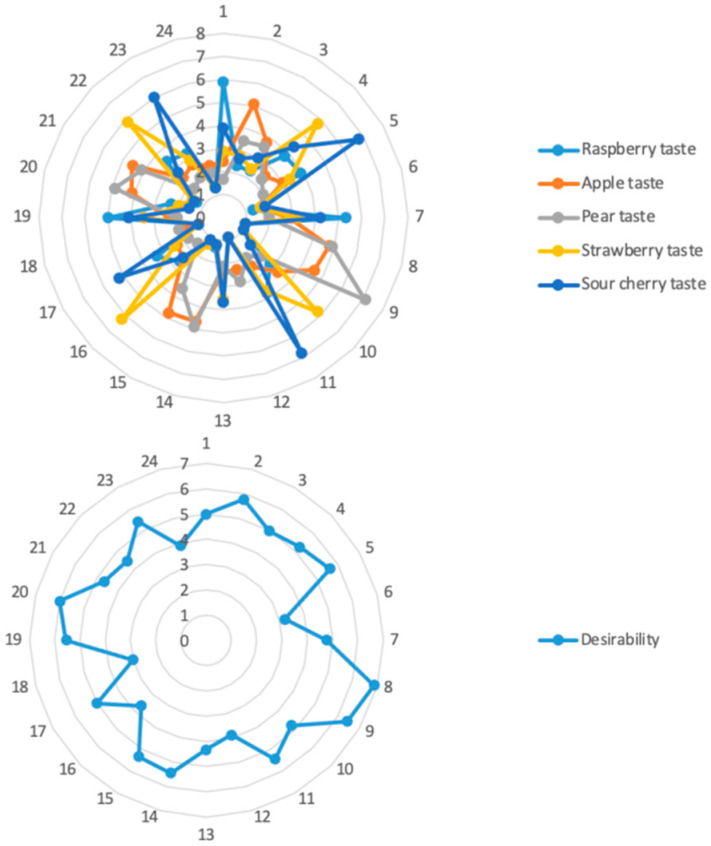
Sensorial characteristics of carrot blended smoothies according to the flavor of the final product and sensorial preferences (1, RJ–PC; 2, AJ–PC; 3, PJ–PC; 4, SJ–PC; 5, SCJ–PC; 6, PC%100; 7, RJ–WC; 8, AJ–WC; 9, PJ–WC; 10, SJ–WC; 11, SCJ–WC; 12, WC%100; 13, RJ–YC; 14, AJ–YC; 15, PJ–YC; 16, SJ–YC; 17, SCJ–YC; 18, YC%100; 19, RJ–OC; 20, AJ–OC; 21, PJ–OC; 22, SJ–OC; 23, SCJ–OC; 24, OC%100).

The sensorial characteristics of the carrot-blended smoothies are presented in Figure 4 and Table 5, including carrot savor and aroma, appearance and sweetness characteristics, raspberry, apple, pear, strawberry or sour cherry flavor, and acceptance of the smoothies.

The panelists evaluated the products for appearance, sweetness, whether they sense the carrot taste, what flavor they sense the most (raspberry, apple, pear, strawberry, or sour cherry flavor), whether they sense the carrot smell, and their desire for the smoothies.

Food appearance depends on the surface color, shape, and size of the food at first sight and determines whether consumers buy or reject it [41]. In the present study, the highest acceptance for the appearances of the smoothies was in the following order: SCJ–WC > RJ–PC > SJ–PC = AJ–WC, whereas the lowest acceptance for the appearance was as follows: SJ–YC < SJ–WC < PJ–PC = SCJ–OC. Thus, PC and WC smoothies showed the highest acceptance for appearance.

Sweet taste is one of the parameters for consumers to purchase a food product. In the present study, the highest sweetness was observed in the following order: AJ–PC = AJ–YC = PJ–YC = AJ–OC, whereas the lowest sweetness was observed as follows: RJ–WC < RJ–PC < RJ–YC. These results show that AJ increases the sugar content in the carrot-blended smoothies, whereas RJ decreases it.

Carrot varieties are rich in nutritional content; however, many people, especially children, do not prefer carrot flavor and aroma in beverages. Therefore, carrot-blended smoothies were evaluated for both carrot taste and smell. The results showed that the highest carrot taste was observed in the following order: PJ–PC > AJ–PC > PJ–OC, whereas the lowest carrot taste was observed as follows: RJ–WC < SCJ–OC < SCJ–PC. Thus, PJ cannot mask the carrot taste, whereas SCJ can easily suppress it. Nevertheless, the highest carrot aroma was observed in the following order: PJ–PC > RJ–PC > AJ–PC, whereas the lowest carrot aroma was observed as follows: SCJ–YC < SJ–YC = SJ–PC. Hence, PC puree samples showed the highest carrot aroma, but the sweet smell of strawberry changed the aroma of the carrot-blended smoothies.

This study showed that with different fruit juices, carrot taste and smell can be suppressed to attract consumption by all age groups. Therefore, the prepared carrot blended smoothies were evaluated for the presence of raspberry, apple, pear, strawberry, or sour cherry taste. As expected, RJ, AJ, PJ, SJ, and SCJ smoothies showed the highest results for taste; however, some extreme results were observed as well. For instance, the PJ–YC smoothie was evaluated as having the taste of apple, and AJ smoothies were evaluated as having the taste of pear. In conclusion, many carrot-blended smoothies were evaluated as not having the flavor and/or aroma of carrot.

Finally, carrot-based smoothies were evaluated for acceptance. The highest acceptance was observed in the following order: AJ–WC > PJ–WC > AJ–OC, whereas the lowest acceptance was observed in the following order: SJ–YC < RJ–YC < SJ–OC. Thus, the AJ–WC smoothie might be used in future beverage preparations.

## 4. Conclusions

This study compared the in vitro biological activities and physicochemical characteristics of different carrot–fruit juice smoothies (raspberry, apple, pear, strawberry, and sour cherry juices). The raspberry juice–purple carrot smoothie showed the highest TA and antioxidant activity (in all assays) against lipase and BuChE inhibitions. These characteristics are not only important from the nutrition perspective but also provide opportunities for beverage processing. The sour cherry juice–purple carrot smoothie showed the highest results for TSS, dry mass, osmolality, total phenolic acid, and total anthocyanin and procyanidin contents. The apple juice–purple carrot smoothie showed the highest carotenoid content. Interestingly, the apple juice–white carrot smoothie was voted for the highest product acceptance, although it did not show potent nutritional content and biological activities. Purple carrot, raspberry, and sour cherry juice smoothies are rich in bioactive compounds with biological activities. The fruit and vegetables studied might be applied together in functional and/or novel food products to improve their nutritional characteristics. On the other hand, bioavailability assays are essential for further analyses to see the biotransformation of the bioactive compounds in carrot-based smoothies during the digestion process. Additionally, shelf-life studies of the obtained smoothies are being conducted, which will answer a number of questions related to the dynamics of changes in the chemical composition and physicochemical properties of aging products.

## Figures and Tables

**Table 1:** Physicochemical parameters of carrot-blended smoothies.

											Color	
Sample	L-Ascorbic Acid	Viscosity	pH	TA	Pectin	TSS	Dry mass	Ash	Osmolality	a	b	L
RJ–PC	155.08 ± 1.16 ^c^	22.75 ± 0.49 ^g^	3.75 ± 0.02 ^ö^	1.21 ± 0.01 ^a^	0.81 ± 0.04 ^defg^	10.20 ± 0.22 ^b^	10.95 ± 0.12 ^j^	0.48 ± 0.12 ^jkl^	534.00 ± 11.33 ^gh^	21.67 ± 0.15 ^e^	11.37 ± 0.26 ^n^	32.21 ± 0.18 ^pr^
AJ–PC	102.21 ± 0.83 ^ghj^	28.37 ± 0.61 ^f^	4.49 ± 0.01 ^f^	0.34 ± 0.00 ^h^	0.58 ± 0.11 ^kl^	12.20 ± 0.26 ^a^	12.80 ± 0.02 ^b^	0.53 ± 0.08 ^j^	683.00 ± 14.49 ^c^	18.80 ± 0.24 ^g^	10.67 ± 0.09 ^ö^	33.83 ± 0.13 ^n^
PJ–PC	109.52 ± 0.80 ^f^	54.08 ± 1.16 ^b^	4.77 ± 0.05 ^d^	0.27 ± 0.01 ^k^	0.80 ± 0.16 ^efgh^	10.90 ± 0.23 ^b^	12.17 ± 0.08 ^d^	0.55 ± 0.02 ^hj^	652.00 ± 13.83 ^cde^	17.19 ± 0.51 ^h^	10.09 ± 0.38 ^p^	34.32 ± 0.40 ^n^
SJ–PC	142.33 ± 2.14 ^d^	47.28 ± 1.02 ^cd^	4.12 ± 0.02 ^j^	0.58 ± 0.02 ^f^	0.88 ± 0.07 ^cde^	8.80 ± 0.19 ^c^	9.52 ± 0.06 ^n^	0.55 ± 0.06 ^hj^	444.00 ± 9.42 ^ij^	19.59 ± 0.50 ^fg^	10.93 ± 0.30 ^ö^	35.04 ± 0.43 ^m^
SCJ–PC	104.67 ± 0.27 ^fghj^	46.98 ± 1.01 ^cd^	4.06 ± 0.03 ^k^	0.82 ± 0.04 ^d^	0.96 ± 0.03 ^cd^	12.40 ± 0.26 ^a^	13.11 ± 0.05 ^a^	0.72 ± 0.09 ^cde^	804.00 ± 17.06 ^a^	16.64 ± 0.62 ^h^	7.98 ± 0.29 ^r^	32.79 ± 0.25 ^ö^
PC%100	91.27 ± 2.18 ^k^	>100.00 ± 0.00 ^a^	5.29 ± 0.02 ^b^	0.23 ± 0.01 ^l^	1.26 ± 0.03 ^a^	10.10 ± 0.21 ^b^	12.60 ± 0.01 ^c^	1.06 ± 0.06 ^a^	663.00 ± 14.06 ^cd^	7.90 ± 0.57 ^l^	5.89 ± 0.18 ^t^	32.74 ± 0.03 ^ö^
RJ–WC	154.09 ± 0.70 ^c^	11.52 ± 0.25 ^jk^	3.62 ± 0.01 ^r^	1.12 ± 0.01 ^b^	0.88 ± 0.05 ^cde^	8.80 ± 0.19 ^c^	9.75 ± 0.01 ^m^	0.49 ± 0.16 ^jk^	548.00 ± 11.62 ^gh^	21.03 ± 0.04 ^e^	8.21 ± 0.01 ^r^	36.77 ± 0.01 ^l^
AJ–WC	101.40 ± 0.91 ^j^	4.43 ± 0.10 ^mn^	4.28 ± 0.03 ^h^	0.34 ± 0.03 ^h^	0.75 ± 0.04 ^ghj^	10.70 ± 0.23 ^b^	11.81 ± 0.07 ^ef^	0.45 ± 0.01 ^kl^	611.00 ± 12.96 ^def^	3.19 ± 0.07 ^p^	19.67 ± 0.07 ^j^	51.78 ± 0.06 ^d^
PJ-WC	173.65 ± 2.98 ^b^	8.85 ± 1.06 ^klm^	4.51 ± 0.01 ^f^	0.31 ± 0.01 ^j^	0.83 ± 0.11 ^def^	10.70 ± 0.23 ^b^	11.97 ± 0.08 ^e^	0.59 ± 0.06 ^fghj^	661.00 ± 14.02 ^cd^	0.57 ± 0.14 ^r^	21.10 ± 0.11 ^h^	55.58 ± 0.47 ^a^
SJ-WC	155.28 ± 0.10 ^c^	30.75 ± 0.64 ^f^	4.05 ± 0.01 ^kl^	0.53 ± 0.04 ^g^	0.50 ± 0.00 ^l^	7.60 ± 0.16 ^de^	8.71 ± 0.17 ^p^	0.61 ± 0.01 ^efghj^	462.00 ± 9.80 ^i^	17.13 ± 0.12 ^h^	16.18 ± 0.08 ^l^	45.19 ± 0.41 ^h^
SCJ-WC	101.51 ± 1.72 ^hj^	6.45 ± 0.64 ^lmn^	3.89 ± 0.01 ^n^	0.83 ± 0.01 ^cd^	0.79 ± 0.13 ^fgh^	10.60 ± 0.22 ^b^	11.89 ± 0.21 ^ef^	0.55 ± 0.12 ^ghj^	690.00 ± 14.64 ^c^	19.98 ± 0.76 ^f^	6.50 ± 0.11 ^s^	31.79 ± 0.59 ^r^
WC%100	91.54 ± 0.54 ^k^	49.05 ± 6.47 ^d^	5.23 ± 0.01 ^c^	0.15 ± 0.01 ^n^	1.03 ± 0.14 ^bc^	7.50 ± 0.16 ^de^	9.19 ± 0.04 ^ö^	0.72 ± 0.00 ^def^	534.00 ± 11.33 ^gh^	4.17 ± 0.04 ^ö^	24.17 ± 0.01 ^f^	53.78 ± 0.03 ^b^
RJ–YC	141.11 ± 0.63 ^d^	36.60 ± 0.85 ^e^	3.74 ± 0.02 ^ö^	1.17 ± 0.02 ^a^	1.10 ± 0.21 ^b^	8.90 ± 0.19 ^c^	10.06 ± 0.04 ^kl^	0.72 ± 0.15 ^defg^	533.00 ± 11.31 ^gh^	21.44 ± 0.25 ^e^	15.22 ± 0.18 ^m^	38.38 ± 0.12 ^k^
AJ–YC	196.76 ± 0.16 ^a^	12.60 ± 1.70 ^jkl^	4.34 ± 0.00 ^g^	0.37 ± 0.02 ^h^	0.76 ± 0.04 ^fgh^	10.20 ± 0.22 ^b^	11.56 ± 0.06 ^g^	0.57 ± 0.03 ^fghj^	625.00 ± 13.26 ^def^	6.51 ± 0.08 ^m^	34.49 ± 0.76 ^d^	51.60 ± 0.21 ^d^
PJ–YC	117.13 ± 0.85 ^e^	17.55 ± 2.23 ^hi^	4.69 ± 0.02 ^e^	0.25 ± 0.01 ^l^	0.73 ± 0.08 ^hjk^	9.80 ± 0.21 ^b^	11.13 ± 0.06 ^hj^	0.58 ± 0.02 ^fghj^	572.00 ± 12.13 ^fgh^	5.49 ± 0.06 ^n^	35.76 ± 0.41 ^c^	53.92 ± 0.16 ^b^
SJ–YC	152.37 ± 3.18 ^c^	49.05 ± 0.21 ^bc^	4.06 ± 0.00 ^k^	0.55 ± 0.00 ^fg^	0.72 ± 0.10 ^hjk^	7.80 ± 0.17 ^de^	8.53 ± 0.11 ^p^	0.74 ± 0.04 ^cd^	446.00 ± 9.46 ^ij^	14.59 ± 0.21 ^j^	23.86 ± 0.30 ^f^	47.28 ± 0.20 ^g^
SCJ–YC	105.35 ± 6.22 ^fgh^	8.85 ± 0.64 ^klm^	3.99 ± 0.00 ^m^	0.85 ± 0.04 ^c^	0.77 ± 0.14 ^fgh^	10.80 ± 0.23 ^b^	12.12 ± 0.09 ^d^	0.84 ± 0.27 ^c^	750.00 ± 15.91 ^b^	19.91 ± 0.46 ^f^	11.20 ± 0.27 ^n^	32.55 ± 0.09 ^öp^
YC%100	76.98 ± 1.68 ^l^	>100.00 ± 0.00 ^a^	5.35 ± 0.00 ^a^	0.24 ± 0.01 ^l^	0.59 ± 0.01 ^jkl^	6.70 ± 0.14 ^f^	9.44 ± 0.16 ^n^	0.94 ± 0.01 ^b^	463.00 ± 9.82 ^i^	8.93 ± 0.02 ^k^	40.47 ± 0.22 ^a^	52.76 ± 0.69 ^c^
RJ–OC	140.22 ± 0.25 ^d^	8.10 ± 0.85 ^klmn^	3.67 ± 0.00 ^p^	1.17 ± 0.00 ^a^	0.92 ± 0.06 ^cde^	8.90 ± 0.19 ^c^	10.14 ± 0.16 ^k^	0.42 ± 0.01 ^klm^	523.00 ± 11.09 ^h^	26.58 ± 0.74 ^b^	22.66 ± 1.15 ^g^	39.63 ± 0.62 ^j^
AJ–OC	171.27 ± 0.04 ^b^	3.30 ± 0.85 ^n^	4.32 ± 0.02 ^g^	0.35 ± 0.01 ^h^	0.26 ± 0.02 ^m^	10.30 ± 0.22 ^b^	11.73 ± 0.04 ^fg^	0.29 ± 0.06 ^mn^	606.00 ± 12.86 ^ef^	25.79 ± 0.17 ^b^	37.11 ± 0.54 ^b^	48.84 ± 0.27 ^f^
PJ–OC	107.35 ± 1.25 ^fg^	4.35 ± 1.06 ^mn^	4.70 ± 0.03 ^de^	0.24 ± 0.01 ^l^	0.75 ± 0.17 ^hj^	9.80 ± 0.21 ^b^	11.14 ± 0.02 ^hj^	0.27 ± 0.01 ^n^	577.00 ± 12.24 ^fg^	26.21 ± 0.23 ^b^	37.54 ± 0.67 ^b^	50.48 ± 0.16 ^e^
SJ–OC	137.34 ± 1.90 ^d^	18.45 ± 0.64 ^gh^	4.04 ± 0.01 ^l^	0.55 ± 0.01 ^f^	0.27 ± 0.03 ^m^	7.40 ± 0.16 ^ef^	8.27 ± 0.04 ^r^	0.30 ± 0.16 ^lmn^	419.00 ± 8.89 ^j^	24.55 ± 0.07 ^c^	29.42 ± 0.19 ^e^	45.15 ± 0.89 ^h^
SCJ–OC	89.13 ± 3.17 ^k^	3.75 ± 0.21 ^mn^	3.91 ± 0.01 ^n^	0.76 ± 0.01 ^e^	0.94 ± 0.03 ^cd^	10.10 ± 0.21 ^b^	11.27 ± 0.21 ^h^	0.40 ± 0.01 ^klm^	662.00 ± 14.04 ^cd^	23.36 ± 0.36 ^d^	17.37 ± 0.43 ^k^	37.18 ± 0.08 ^l^
OC%100	80.05 ± 0.62 ^l^	15.15 ± 1.48 ^hi^	5.33 ± 0.00 ^ab^	0.20 ± 0.02 ^m^	nd	8.20 ± 0.17 ^cd^	9.97 ± 0.04 ^lm^	0.63 ± 0.07 ^defgh^	459.00 ± 9.74 ^i^	28.70 ± 0.09 ^a^	36.67 ± 0.34 ^b^	50.47 ± 0.10 ^e^

L-ascorbic acid (mg/100 mL); Viscosity [mPas]; TA (g malic acid/100 mL); Pectin [%]; TSS, Total Soluble Solids [°Brix]; Dry mass [%]; Ash [%]; Osmolality [mOsm/liter]; significant at *p* ≤ 0.05; values (mean of three replications) followed by the same letter within the same column were not significantly different (*p* ≤ 0.05) according to Tukey’s test; not detected (nd).

**Table 2:** The mineral contents of carrot-based smoothies.

Sample	Na	K	Ca	Fe	Mg
RJ–PC	20.65 ± 0.44 ^k^	172.72 ± 3.66 ^def^	2.86 ± 0.06 ^f^	0.41 ± 0.01 ^c^	0.47 ± 0.01 ^cd^
AJ–PC	29.81 ± 0.63 ^fg^	159.00 ± 3.37 ^fg^	4.79 ± 0.10 ^c^	0.47 ± 0.01 ^b^	1.14 ± 0.02 ^b^
PJ–PC	24.70 ± 0.52 ^hj^	163.02 ± 3.46 ^efg^	2.66 ± 0.06 ^fgh^	0.26 ± 0.01 ^f^	0.45 ± 0.01 ^cd^
SJ–PC	23.84 ± 0.51 ^j^	161.53 ± 3.43 ^fg^	2.73 ± 0.06 ^fg^	0.26 ± 0.01 ^f^	0.45 ± 0.01 ^cd^
SCJ–PC	33.22 ± 0.70 ^f^	174.54 ± 3.70 ^cdef^	5.33 ± 0.11 ^b^	0.42 ± 0.01 ^c^	1.27 ± 0.03 ^a^
PC%100	59.25 ± 1.26 ^b^	326.20 ± 6.92 ^a^	8.18 ± 0.17 ^a^	0.63 ± 0.01 ^a^	1.28 ± 0.03 ^a^
RJ–WC	27.22 ± 0.58 ^gh^	145.19 ± 3.08 ^g^	2.38 ± 0.05 ^hj^	0.24 ± 0.01 ^f^	0.42 ± 0.01 ^d^
AJ–WC	21.70 ± 0.46 ^k^	115.33 ± 2.45 ^kl^	2.18 ± 0.05 ^j^	0.19 ± 0.00 ^j^	0.46 ± 0.01 ^cd^
PJ–WC	25.96 ± 0.55 ^h^	129.30 ± 2.74 ^hj^	2.43 ± 0.05 ^ghj^	0.23 ± 0.00 ^fg^	0.42 ± 0.01 ^d^
SJ–WC	27.25 ± 0.58 ^gh^	131.92 ± 2.80 ^hj^	2.40 ± 0.05 ^ghj^	0.20 ± 0.00 ^j^	0.39 ± 0.01 ^def^
SCJ–WC	25.65 ± 0.54 ^hj^	172.04 ± 3.65 ^def^	2.19 ± 0.05 ^j^	0.19 ± 0.00 ^j^	0.44 ± 0.01 ^cd^
WC%100	41.50 ± 0.88 ^d^	190.73 ± 4.05 ^c^	3.66 ± 0.08 ^e^	0.26 ± 0.01 ^f^	0.44 ± 0.01 ^cd^
RJ–YC	52.49 ± 1.11 ^c^	177.98 ± 3.78 ^cde^	5.44 ± 0.12 ^b^	0.49 ± 0.01 ^b^	1.16 ± 0.02 ^b^
AJ–YC	38.78 ± 0.82 ^de^	137.59 ± 2.92 ^gh^	2.72 ± 0.06 ^fg^	0.20 ± 0.00 ^hj^	0.41 ± 0.01 ^de^
PJ–YC	37.76 ± 0.80 ^e^	139.22 ± 2.95 ^gh^	2.69 ± 0.06 ^fgh^	0.23 ± 0.00 ^fg^	0.42 ± 0.01 ^d^
SJ–YC	36.80 ± 0.78 ^e^	148.53 ± 3.15 ^g^	2.86 ± 0.06 ^f^	0.25 ± 0.01 ^f^	0.42 ± 0.01 ^d^
SCJ–YC	37.07 ± 0.79 ^e^	209.42 ± 4.44 ^b^	2.45 ± 0.05 ^ghj^	0.35 ± 0.01 ^d^	0.42 ± 0.01 ^d^
YC%100	68.33 ± 1.45 ^a^	222.04 ± 4.71 ^b^	4.24 ± 0.09 ^d^	0.31 ± 0.01 ^e^	0.37 ± 0.01 ^f^
RJ–OC	25.59 ± 0.54 ^hj^	158.22 ± 3.36 ^fg^	2.10 ± 0.04 ^j^	0.25 ± 0.01 ^f^	0.40 ± 0.01 ^de^
AJ–OC	23.74 ± 0.50 ^j^	125.23 ± 2.66 ^jk^	1.68 ± 0.04 ^l^	0.20 ± 0.00 ^hj^	0.38 ± 0.01 ^ef^
PJ–OC	25.59 ± 0.54 ^hj^	114.40 ± 2.43 ^kl^	1.59 ± 0.03 ^l^	0.22 ± 0.00 ^gh^	0.42 ± 0.01 ^d^
SJ–OC	21.36 ± 0.45 ^k^	108.20 ± 2.30 ^l^	2.11 ± 0.04 ^j^	0.26 ± 0.01 ^f^	0.41 ± 0.01 ^de^
SCJ–OC	24.26 ± 0.51 ^hj^	165.65 ± 3.51 ^def^	1.90 ± 0.04 ^k^	0.26 ± 0.01 ^f^	0.42 ± 0.01 ^d^
OC%100	39.46 ± 0.84 ^de^	180.58 ± 3.83 ^cd^	3.53 ± 0.07 ^e^	0.32 ± 0.01 ^e^	0.49 ± 0.01 ^c^

The content of minerals (mg/100 mL) in carrot-blended smoothies, significant at *p* ≤ 0.05; values (mean of three replications) followed by the same letter within the same column were not significantly different (*p* ≤ 0.05) according to Tukey’s test.

**Table 3:** Identified polyphenolics by LC/MS in carrot-based smoothies.

							Purple Carrot						White Carrot						Yellow Carrot						Orange Carrot				
	Compound	Rt (min)	λmax (nm)	MS [M–H]− (m/z)	MS/ MS (m/z)	PC	RJ	AJ	PJ	SJ	SCJ	WC	RJ	AJ	PJ	SJ	SCJ	YC	RJ	AJ	PJ	SJ	SCJ	OC	RJ	AJ	PJ	SJ	SCJ
Flavan-3-ols	procyanidin B2	4.26	280	577	289	+	+	+	+	+	+	+	+	+	+	+	+	+	+	+	+	+	nd	+	nd	nd	nd	+	+
	procyanidin B4	3.92	279	577	289	+	+	nd	+	+	nd	nd	+	nd	nd	nd	nd	nd	+	nd	nd	+	+	nd	+	nd	nd	+	+
	epicatechin	4.74	278	289	289	+	+	nd	nd	nd	nd	nd	nd	nd	nd	nd	nd	nd	nd	nd	nd	nd	nd	nd	nd	nd	nd	nd	nd
Phenolic acids	3-O-caffeoylquinic acid	5.90	324	353	135/179/191	+	+	+	+	+	+	nd	nd	nd	nd	nd	+	nd	nd	nd	nd	nd	+	nd	nd	nd	nd	nd	+
	chlorogenic acid	3.86	326	353	191	+	+	nd	+	+	nd	+	+	+	+	nd	+	+	nd	+	nd	nd	nd	+	+	+	nd	nd	+
	5-O-caffeoylquinic acid	7.54	325	353	179/191	+	+	+	+	+	+	+	+	+	+	+	+	+	+	+	+	+	+	+	+	+	+	+	+
	4-O-caffeoylquinic acid	8.03	325	353	179/191	+	+	+	+	+	+	nd	+	+	nd	+	nd	nd	nd	nd	nd	+	+	nd	nd	nd	nd	+	+
	ferulic acid-hexoside	8.89	325	355	193/175	+	+	+	+	nd	+	+	+	+	+	+	+	+	+	nd	nd	nd	nd	+	+	+	+	+	+
	ferulic acid di-hexoside	9.41	324	517	355/193/175	+	+	+	+	+	+	nd	nd	nd	nd	nd	nd	nd	nd	nd	nd	nd	nd	nd	nd	nd	nd	nd	nd
	3-O-feruloylquinic acid	10.10	322	367	173/193	nd	+	+	+	+	+	+	+	+	+	+	+	nd	nd	nd	nd	nd	nd	nd	nd	nd	nd	nd	nd
	4-O-feruloylquinic acid	10.84	323	367	173/193	+	+	+	+	+	+	+	+	+	+	+	+	+	+	+	+	+	+	+	+	+	+	+	+
	caffeic acid -hexoside	10.90	324	341	179/135	+	+	+	+	+	+	nd	nd	nd	nd	nd	nd	nd	nd	nd	nd	nd	nd	nd	nd	nd	nd	nd	nd
	di-ferulic acid derivative	14.27	327	527	203/365/366	+	+	+	+	+	+	+	+	+	+	+	+	+	+	+	+	+	+	+	+	+	+	+	+
	dicaffeoylquinic acid	7.01	320	515	353/191	+	nd	+	nd	nd	nd	nd	nd	nd	nd	nd	nd	nd	nd	nd	nd	nd	nd	nd	nd	nd	nd	nd	nd
	cis-5-p-coumaroylquinic acid	6.32	320	337	163/191	+	nd	nd	nd	nd	+	nd	nd	nd	nd	nd	nd	nd	nd	nd	nd	nd	nd	nd	nd	nd	nd	nd	nd
	*p*-coumaric acid	4.87	312	325	163/119	nd	nd	nd	nd	nd	+	nd	nd	nd	nd	nd	nd	nd	nd	nd	nd	nd	nd	nd	nd	nd	nd	nd	nd
Flavanols	quercetin-3-galactoside	7.71	354	609	447/301	nd	+	nd	nd	+	+	nd	+	nd	nd	+	+	nd	+	nd	nd	+	+	nd	+	nd	nd	+	+
	genistin	6.86	326	269	133	nd	nd	nd	nd	nd	+	nd	nd	nd	nd	nd	+	nd	nd	nd	nd	nd	+	nd	nd	nd	nd	nd	+
Anthocyanins	cyanidin-3-O-xylosyl -glucosylgalactoside	5.58	517	743 ^+^	287	+	+	+	+	+	+	nd	nd	nd	nd	nd	nd	nd	nd	nd	nd	nd	nd	nd	nd	nd	nd	nd	nd
	cyanidin-3-O -xylosyl-galactoside	6.14	518	581 ^+^	287	nd	+	nd	nd	nd	+	nd	+	nd	nd	nd	+	nd	+	nd	nd	nd	+	nd	+	nd	nd	nd	+
	cyanidin-3-O-xylosyl -cinpoyl-glucosylgalactoside	7.22	530	949 ^+^	287	+	+	+	+	+	+	nd	nd	nd	nd	nd	+	nd	nd	nd	nd	nd	nd	nd	nd	nd	nd	nd	nd
	cyanidin-3-O-xylosyl -feruloyl-glucosylgalactoside	7.57	528	919 ^+^	287	+	nd	+	+	+	+	nd	nd	nd	nd	nd	nd	nd	nd	nd	nd	nd	nd	nd	nd	nd	nd	nd	nd
	cyanidin-3-O-xylosyl -p-coumaroylglucosyl-galactoside	7.68	527	889 ^+^	287	+	+	+	+	+	+	nd	nd	nd	nd	+	nd	nd	nd	nd	nd	+	nd	nd	nd	nd	nd	+	nd
	cyanidin-3-O -glucosyl-rutinoside	5.98	520	757 ^+^	611/297	nd	nd	nd	nd	nd	+	nd	+	nd	nd	nd	nd	nd	+	nd	nd	nd	+	nd	+	nd	nd	nd	+
	cyanidin-3-arabinoside	5.11	520	419 ^+^	287	nd	nd	nd	nd	nd	nd	nd	+	nd	nd	nd	nd	nd	+	nd	nd	nd	nd	nd	+	nd	nd	nd	nd

not detected (nd); +—for anthocyanins positive mode (MS [M–H]^+^).

**Table 4:** The antioxidant [mmol TE/100 mL] and in vitro inhibition activities (acetylcholinesterase, butyrylcholinesterase, α-amylase, α-glucosidase, pancreatic lipase) of carrot-based smoothies.

Sample	ABTS	FRAP	ORAC	α-Amylase [IC50]	α-Glucosidase [IC50]	Lipase [IC50]	AChE [% inh]	BuChE [% inh]
RJ–PC	1.78 ± 0.31 ^a^	1.47 ± 0.03 ^a^	0.42 ± 0.02 ^a^	543.86 ± 11.54 ^ghj^	278.78 ± 5.91 ^hj^	3.18 ± 0.07 ^r^	9.77 ± 0.21 ^b^	21.13 ± 0.45 ^a^
AJ–PC	1.03 ± 0.05 ^hjk^	0.87 ± 0.01 ^jk^	0.12 ± 0.00 ^f^	339.98 ± 7.21 ^mn^	2966.52 ± 62.93 ^a^	11.14 ± 0.24 ^h^	6.53 ± 0.14 ^gh^	14.41 ± 0.31 ^bc^
PJ–PC	1.06 ± 0.06 ^gh^	0.96 ± 0.02 ^gh^	0.10 ± 0.01 ^fg^	472.06 ± 10.01 ^hj^	834.76 ± 17.71 ^c^	10.85 ± 0.23 ^hj^	5.16 ± 0.11 ^kl^	13.33 ± 0.28 ^c^
SJ–PC	1.71 ± 0.02 ^ab^	1.32 ± 0.04 ^b^	0.13 ± 0.01 ^e^	312.44 ± 6.63 ^n^	729.38 ± 15.47 ^d^	7.94 ± 0.17 ^l^	8.01 ± 0.17 ^cd^	15.23 ± 0.32 ^b^
SCJ–PC	1.67 ± 0.21 ^b^	1.25 ± 0.05 ^c^	0.22 ± 0.02 ^c^	350.34 ± 7.43 ^lm^	312.01 ± 6.62 ^ghj^	3.70 ± 0.08 ^p^	4.84 ± 0.10 ^lm^	11.01 ± 0.23 ^d^
PC%100	1.51 ± 0.47 ^c^	1.08 ± 0.04 ^ef^	0.28 ± 0.04 ^b^	374.94 ± 7.95 ^kl^	3020.95 ± 64.08 ^a^	15.17 ± 0.32 ^efg^	4.88 ± 0.10 ^lm^	5.17 ± 0.11 ^h^
RJ–WC	0.98 ± 0.03 ^jk^	0.99 ± 0.02 ^g^	0.06 ± 0.01 ^k^	445.34 ± 9.45 ^hj^	182.99 ± 3.88 ^k^	5.23 ± 0.11 ^o^	7.22 ± 0.15 ^ef^	14.37 ± 0.30 ^bc^
AJ–WC	0.58 ± 0.06 ^m^	0.55 ± 0.02 ^nö^	0.05 ± 0.00 ^l^	752.39 ± 15.96 ^f^	680.19 ± 14.43 ^de^	14.12 ± 0.30 ^g^	5.83 ± 0.12 ^j^	6.41 ± 0.14 ^g^
PJ–WC	0.81 ± 0.05 ^l^	0.80 ± 0.04 ^lm^	0.03 ± 0.00 ^n^	2378.99 ± 50.47 ^b^	618.14 ± 13.11 ^e^	16.18 ± 0.34 ^cde^	4.50 ± 0.10 ^m^	4.27 ± 0.09 ^l^
SJ–WC	1.17 ± 0.17 ^ef^	1.13 ± 0.02 ^e^	0.09 ± 0.01 ^ghj^	391.47 ± 8.30 ^jk^	158.57 ± 3.36 ^l^	5.93 ± 0.13 ^m^	5.96 ± 0.13 ^hj^	9.03 ± 0.19 ^ef^
SCJ–WC	1.04 ± 0.06 ^ghj^	0.84 ± 0.02 ^kl^	0.16 ± 0.00 ^d^	1907.24 ± 40.46 ^c^	287.00 ± 6.09 ^hj^	8.64 ± 0.18 ^kl^	4.85 ± 0.10 ^lm^	9.98 ± 0.21 ^de^
WC%100	0.40 ± 0.02 ^p^	0.41 ± 0.01 ^r^	0.01 ± 0.00 ^ö^	539.63 ± 11.45 ^ghj^	353.09 ± 7.49 ^gh^	20.76 ± 0.44 ^a^	5.14 ± 0.11 ^kl^	3.53 ± 0.07 ^m^
RJ–YC	1.12 ± 0.01 ^fg^	1.10 ± 0.04 ^e^	0.06 ± 0.01 ^k^	995.64 ± 21.12 ^e^	290.58 ± 6.16 ^hj^	5.32 ± 0.11 ^no^	8.09 ± 0.17 ^c^	21.03 ± 0.45 ^a^
AJ–YC	1.03 ± 0.06 ^hjk^	0.91 ± 0.03 ^hj^	0.05 ± 0.00 ^l^	548.30 ± 11.63 ^gh^	495.43 ± 10.51 ^f^	14.65 ± 0.31 ^fg^	2.96 ± 0.06 ^o^	4.44 ± 0.09 ^kl^
PJ–YC	0.42 ± 0.12 ^öp^	0.53 ± 0.02 ^öp^	0.04 ± 0.00 ^m^	663.66 ± 14.08 ^fg^	867.60 ± 18.40 ^c^	16.90 ± 0.36 ^bc^	4.59 ± 0.10 ^lm^	4.84 ± 0.10 ^hj^
SJ–YC	1.21 ± 0.08 ^e^	1.04 ± 0.01 ^f^	0.06 ± 0.00 ^k^	1759.15 ± 37.32 ^c^	311.45 ± 6.61 ^ghj^	9.78 ± 0.21 ^jk^	7.54 ± 0.16 ^cde^	6.46 ± 0.14 ^g^
SCJ–YC	0.76 ± 0.09 ^l^	0.38 ± 0.02 ^r^	0.16 ± 0.01 ^d^	338.59 ± 7.18 ^mn^	172.95 ± 3.67 ^kl^	5.44 ± 0.12 ^no^	10.77 ± 0.23 ^a^	20.72 ± 0.44 ^a^
YC%100	0.32 ± 0.03 ^r^	0.36 ± 0.00 ^r^	0.04 ± 0.00 ^m^	3772.45 ± 80.03 ^a^	1060.69 ± 22.50 ^b^	16.53 ± 0.35 ^cd^	7.10 ± 0.15 ^efg^	4.63 ± 0.10 ^jkl^
RJ–OC	1.11 ± 0.08 ^fgh^	1.12 ± 0.04 ^e^	0.08 ± 0.01 ^j^	717.65 ± 15.22 ^f^	126.09 ± 2.67 ^m^	5.71 ± 0.12 ^mn^	6.94 ± 0.15 ^fg^	14.39 ± 0.31 ^bc^
AJ–OC	0.93 ± 0.08 ^k^	0.87 ± 0.03 ^k^	0.05 ± 0.00 ^l^	1859.88 ± 39.45 ^c^	188.08 ± 3.99 ^k^	15.57 ± 0.33 ^def^	5.48 ± 0.12 ^jk^	4.69 ± 0.10 ^jk^
PJ–OC	0.48 ± 0.05 ^nö^	0.59 ± 0.02 ^n^	0.03 ± 0.00 ^n^	1198.85 ± 25.43 ^d^	658.98 ± 13.98 ^de^	17.93 ± 0.38 ^b^	2.45 ± 0.05 ^p^	4.94 ± 0.10 ^hj^
SJ–OC	1.36 ± 0.11 ^d^	1.19 ± 0.08 ^d^	0.10 ± 0.03 ^fg^	419.00 ± 8.89 ^j^	247.10 ± 5.24 ^j^	8.96 ± 0.19 ^kl^	3.81 ± 0.08 ^n^	7.22 ± 0.15 ^g^
SCJ–OC	1.39 ± 0.06 ^d^	0.74 ± 0.10 ^m^	0.27 ± 0.03 ^b^	1066.91 ± 22.63 ^e^	282.21 ± 5.99 ^hj^	7.75 ± 0.16 ^l^	7.43 ± 0.16 ^def^	8.67 ± 0.18 ^f^
OC%100	0.53 ± 0.00 ^mn^	0.49 ± 0.01 ^p^	0.09 ± 0.00 ^ghj^	1087.83 ± 23.08 ^de^	401.47 ± 8.52 ^g^	16.67 ± 0.35 ^cd^	5.02 ± 0.11 ^klm^	4.43 ± 0.09 ^kl^

TE, Trolox equivalents. Significant at *p* ≤ 0.05; values (mean of three replications) followed by the same letter within the same column were not significantly different (*p* ≤ 0.05) according to Tukey’s test.

**Table 5:** Sensorial characteristics of carrot blended smoothies.

	Appearance	Sweetness	Carrot Taste	Carrot Smell
RJ–VC	7.77 ± 0.16 ^ab^	2.77 ± 0.06 ^h^	4.55 ± 0.10 ^jk^	5.88 ± 0.12 ^ef^
AJ–VC	6.77 ± 0.14 ^efg^	6.88 ± 0.15 ^a^	6.55 ± 0.14 ^de^	5.55 ± 0.12 ^efg^
PJ–VC	6.11 ± 0.13 ^g^	6.00 ± 0.13 ^b^	7.00 ± 0.15 ^d^	6.00 ± 0.13 ^de^
SJ–VC	7.55 ± 0.16 ^bc^	3.22 ± 0.07 ^fg^	3.33 ± 0.07 ^no^	3.33 ± 0.07 ^n^
SCJ–VC	6.88 ± 0.15 ^defg^	3.55 ± 0.08 ^de^	3.22 ± 0.07 ^o^	4.11 ± 0.09 ^kl^
VC%100	2.22 ± 0.05 ^m^	3.66 ± 0.08 ^d^	8.11 ± 0.17 ^b^	9.00 ± 0.19 ^a^
RJ–WC	7.44 ± 0.16 ^bcd^	2.44 ± 0.05 ^j^	2.66 ± 0.06 ^p^	3.66 ± 0.08 ^lm^
AJ–WC	7.55 ± 0.16 ^bc^	6.66 ± 0.14 ^a^	5.22 ± 0.11 ^gh^	5.44 ± 0.12 ^fg^
PJ–WC	7.33 ± 0.16 ^bcde^	6.55 ± 0.14 ^a^	4.33 ± 0.09 ^kl^	3.77 ± 0.08 ^lm^
SJ–WC	5.00 ± 0.11 ^j^	3.55 ± 0.08 ^de^	4.11 ± 0.09 ^kl^	4.00 ± 0.08 ^l^
SCJ–WC	8.22 ± 0.17 ^a^	3.33 ± 0.07 ^ef^	3.33 ± 0.07 ^no^	3.55 ± 0.08 ^mn^
WC%100	5.66 ± 0.12 ^h^	5.00 ± 0.11 ^c^	8.66 ± 0.18 ^a^	7.77 ± 0.16 ^b^
RJ–YC	6.33 ± 0.13 ^g^	3.00 ± 0.06 ^gh^	4.22 ± 0.09 ^kl^	4.22 ± 0.09 ^jkl^
AJ–YC	6.88 ± 0.15 ^defg^	6.88 ± 0.15 ^a^	5.00 ± 0.11 ^hj^	3.88 ± 0.08 ^l^
PJ–YC	6.55 ± 0.14 ^fg^	6.88 ± 0.15 ^a^	4.33 ± 0.09 ^kl^	3.55 ± 0.08 ^mn^
SJ–YC	4.33 ± 0.09 ^k^	3.66 ± 0.08 ^d^	3.66 ± 0.08 ^m^	3.33 ± 0.07 ^n^
SCJ–YC	7.33 ± 0.16 ^bcde^	4.11 ± 0.09 ^d^	4.00 ± 0.08 ^l^	3.00 ± 0.06 ^o^
YC%100	3.11 ± 0.07 ^l^	4.77 ± 0.10 ^c^	7.66 ± 0.16 ^bc^	6.88 ± 0.15 ^c^
RJ–OC	6.44 ± 0.14 ^fg^	3.66 ± 0.08 ^d^	3.55 ± 0.08 ^mn^	3.66 ± 0.08 ^lm^
AJ–OC	7.00 ± 0.15 ^cdef^	6.88 ± 0.15 ^a^	5.66 ± 0.12 ^fg^	4.55 ± 0.10 ^jk^
PJ–OC	6.88 ± 0.15 ^defg^	6.77 ± 0.14 ^a^	6.11 ± 0.13 ^ef^	5.11 ± 0.11 ^gh^
SJ–OC	6.33 ± 0.13 ^g^	4.00 ± 0.08 ^d^	3.66 ± 0.08 ^m^	3.88 ± 0.08 ^l^
SCJ–OC	6.11 ± 0.13 ^g^	4.00 ± 0.08 ^d^	2.77 ± 0.06 ^p^	4.66 ± 0.10 ^hj^
OC%100	5.11 ± 0.11 ^j^	5.66 ± 0.12 ^b^	7.55 ± 0.16 ^c^	6.44 ± 0.14 ^cd^

Significant at *p* ≤ 0.05; values (mean of three replications) followed by the same letter within the same column were not significantly different (*p* ≤ 0.05) according to Tukey’s test.

## Data Availability

Data is contained within the article and Supplementary Material (Supplementary S1: Polyphenolic and carotenoid contents of carrot-based smoothies).

## Supplementary Materials

The following supporting information can be downloaded at: https://www.mdpi.com/article/10.3390/antiox12040917/s1, Supplementary S1: Polyphenolic and carotenoid contents of carrot-based smoothies.
