# Supplementary material for: Fruit–Carrot-Based Smoothies as Innovative Products with a Complex Matrix of Bioactive Compounds Effected on Activities of Selected Digestive Enzymes and Cholinesterases In Vitro
Source: Antioxidants (Basel). 2023 Apr 12;12(4):917. doi: 10.3390/antiox12040917 (PMC10135636; doi:10.3390/antiox12040917)
Supplement: Supplementary file 1 [file antioxidants-12-00917-s001.zip › antioxidants-2280705-SI.pdf]

Supplementary S1. Polyphenolic and carotenoid contents of carrot-based smoothies

Sample	Fl3 1	Fl3 2	Fl3 3	Other Fl3	Total Fl3	PA 1	PA 2	PA 3	PA 4	PA 5	PA 6	PA 7	PA 8	PA 9	PA 10	PA 11	P 12	P 13	Other PA	Total PA
RJ-PC	26.23±0.56^{b}	2.37±0.05^{cde}	41.41±0.88^{b}	14.07±0.30^{f}	**84.08±1.78^{d}**	1.05±0.02^{c}	35.89±0.76^{d}	4.59±0.10^{c}	2.75±0.06^{d}	2.40±0.05^{d}	1.58±0.03^{fg}	0.88±0.02^{d}	2.16±0.05^{hj}	nd	nd	nd	nd	nd	nd	**58.65±2.24^{e}**
AJ-PC	18.63±0.40^{de}	nd	nd	33.43±0.71^{j}	**52.06±1.10^{h}**	nd	43.54±0.92^{b}	5.15±0.11^{b}	3.96±0.08^{b}	2.58±0.05^{cd}	1.49±0.03^{g}	0.99±0.02^{bc}	2.68±0.06^{f}	1.72±0.04^{b}	nd	nd	nd	nd	1.24±0.03^{ef}	**71.89±1.52^{c}**
PJ-PC	18.04±0.38^{e}	2.22±0.05^{de}	nd	9.41±0.20^{m}	**29.66±0.63^{n}**	1.07±0.02^{c}	40.37±0.86^{c}	4.09±0.09^{d}	4.02±0.09^{b}	2.78±0.06^{b}	1.65±0.04^{ef}	1.08±0.02^{b}	2.28±0.05^{ghj}	nd	nd	nd	nd	nd	2.11±0.04^{c}	**68.30±1.45^{cd}**
SJ-PC	18.92±0.40^{de}	4.12±0.09^{cd}	nd	18.30±0.39^{b}	**41.34±0.88^{kl}**	1.23±0.03^{b}	35.44±0.75^{d}	4.64±0.10^{c}	3.43±0.07^{c}	2.85±0.06^{b}	1.77±0.04^{de}	0.96±0.02^{cd}	2.46±0.05^{fgh}	nd	nd	nd	nd	nd	3.37±0.07^{a}	**63.92±3.36^{d}**
SCJ-PC	19.92±0.42^{d}	nd	nd	17.39±0.37^{hj}	**37.32±0.79^{lm}**	nd	39.66±0.84^{c}	5.35±0.11^{b}	3.10±0.07^{cd}	2.75±0.06^{bc}	1.61±0.03^{fg}	0.91±0.02^{cd}	2.56±0.05^{fg}	nd	5.21±0.11^{a}	0.99±0.02^{a}	nd	nd	1.64±0.03^{d}	**80.54±2.71^{b}**
PC%100	35.73±0.76^{a}	4.71±0.10^{c}	58.60±1.24^{a}	nd	**99.04±2.10^{c}**	1.94±0.04^{a}	72.18±1.53^{a}	12.13±0.26^{a}	7.61±0.16^{a}	5.82±0.12^{a}	3.20±0.07^{a}	2.35±0.05^{a}	5.21±0.11^{b}	3.78±0.08^{a}	0.36±0.01^{b}	nd	nd	1.15±0.02^{a}	2.68±0.06^{b}	**140.27±2.98^{a}**
RJ-WC	9.08±0.19^{j}	25.04±0.53^{b}	nd	26.54±0.57^{f}	**60.66±1.29^{g}**	0.65±0.01^{e}	2.77±0.06^{n}	0.95±0.02^{ef}	nd	0.33±0.01^{g}	0.80±0.02^{m}	nd	1.24±0.03^{l}	nd	nd	nd	0.30±0.01^{a}	nd	nd	**14.40±0.31^{n}**
AJ-WC	7.46±0.16^{kl}	nd	nd	34.37±0.73^{j}	**41.83±0.89^{kl}**	0.52±0.01^{f}	9.03±0.19^{hj}	0.47±0.01^{g}	nd	0.30±0.01^{g}	0.88±0.02^{l}	nd	1.54±0.03^{k}	nd	nd	nd	nd	nd	nd	**21.13±0.45^{l}**
PJ-WC	8.82±0.19^{jk}	nd	nd	11.06±0.23^{hjkl}	**19.89±0.42^{p}**	0.66±0.01^{e}	11.85±0.25^{fg}	nd	nd	0.39±0.01^{fg}	1.15±0.02^{hj}	nd	2.07±0.04^{j}	nd	nd	nd	nd	nd	nd	**25.65±0.54^{k}**
SJ-WC	12.35±0.26^{g}	nd	nd	20.85±0.44^{b}	**33.21±0.70^{m}**	nd	3.94±0.08^{l}	1.18±0.03^{e}	nd	0.34±0.01^{fg}	1.27±0.03^{h}	nd	2.05±0.04^{j}	nd	nd	nd	nd	nd	1.17±0.02^{f}	**20.79±0.44^{lm}**
SCJ-WC	10.00±0.21^{hj}	nd	nd	60.84±1.29^{b}	**70.84±1.50^{ef}**	0.79±0.02^{d}	7.29±0.15^{j}	nd	nd	0.38±0.01^{fg}	1.06±0.02^{jk}	nd	2.20±0.05^{hj}	nd	nd	nd	nd	nd	nd	**28.99±0.62^{k}**
WC%100	16.29±0.35^{f}	nd	nd	9.17±0.19^{hj}	**25.46±0.54^{ö}**	1.11±0.02^{c}	5.20±0.11^{k}	nd	nd	0.67±0.01^{e}	2.02±0.04^{b}	0.28±0.01^{e}	3.08±0.07^{e}	nd	nd	nd	nd	nd	nd	**25.01±0.53^{k}**
RJ-YC	17.75±0.38^{e}	23.86±0.51^{b}	nd	31.87±0.67^{e}	**73.48±1.56^{e}**	nd	3.36±0.07^{m}	nd	nd	nd	0.95±0.02^{kl}	nd	1.08±0.02^{m}	nd	nd	nd	nd	nd	nd	**19.16±0.41^{m}**
AJ-YC	11.15±0.24^{gh}	nd	nd	33.38±0.70^{j}	**44.53±0.94^{jk}**	0.71±0.02^{de}	8.57±0.18^{hj}	nd	nd	nd	0.81±0.02^{m}	nd	0.95±0.02^{n}	nd	nd	nd	nd	nd	nd	**25.04±0.53^{k}**
PJ-YC	11.49±0.24^{g}	nd	nd	13.55±0.29^{j}	**25.07±0.53^{ö}**	nd	10.02±0.21^{gh}	nd	nd	nd	0.76±0.02^{m}	nd	0.87±0.02^{n}	nd	nd	nd	nd	nd	nd	**26.65±0.57^{k}**
SJ-YC	15.12±0.32^{f}	1.67±0.04^{e}	nd	42.88±1.06^{d}	**66.44±1.41^{fg}**	nd	2.04±0.04^{ö}	1.01±0.02^{ef}	nd	nd	0.68±0.01^{n}	nd	0.69±0.01^{ö}	nd	nd	nd	nd	nd	1.27±0.03^{ef}	**21.70±0.46^{l}**
SCJ-YC	nd	55.92±1.19^{a}	nd	57.29±1.22^{c}	**113.22±2.40^{b}**	nd	5.51±0.12^{k}	0.86±0.02^{ef}	nd	nd	0.61±0.01^{ö}	nd	0.66±0.01^{ö}	nd	nd	nd	nd	nd	nd	**30.39±0.64^{jk}**
YC%100	23.36±0.50^{c}	nd	nd	6.48±0.14^{h}	**29.84±0.63^{n}**	1.91±0.04^{a}	5.48±0.12^{k}	nd	nd	0.53±0.01^{ef}	1.86±0.04^{cd}	nd	1.98±0.04^{j}	nd	nd	nd	nd	nd	nd	**29.76±0.63^{k}**
RJ-OC	nd	24.86±0.	nd	25.13±0.54^{g}	**50.00±1.06^{hj}**	0.70±0.0	8.82±0.19	nd	nd	nd	0.89±0.0	nd	4.51±0.10	nd	nd	nd	nd	nd	nd	**34.92±0.74^{hj}**

		53^{b}				1^{de}	^{hj}				2^{l}		^{d}							
AJ-OC	nd	nd	nd	66.68±0.78^{j}	**36.68±0.78^{lm}**	0.62±0.01^{e}	13.73±0.29^{ef}	nd	nd	nd	0.94±0.02^{kl}	nd	4.75±0.10^{cd}	nd	nd	nd	nd	nd	nd	**40.51±0.86^{g}**
PJ-OC	nd	nd	nd	12.14±0.26^{ghj}	**12.14±0.26^{s}**	nd	15.27±0.32^{e}	nd	nd	nd	0.98±0.02^{kl}	nd	4.74±0.10^{cd}	nd	nd	nd	nd	nd	nd	**42.54±0.90^{fg}**
SJ-OC	5.31±0.11^{mn}	2.84±0.06^{cde}	nd	28.59±0.60^{a}	**36.74±0.78^{lm}**	nd	7.41±0.16^{j}	1.10±0.02^{e}	nd	nd	1.06±0.02^{jk}	nd	4.74±0.10^{cd}	nd	nd	nd	nd	nd	1.35±0.03^{e}	**38.63±0.82^{gh}**
SCJ-OC	4.39±0.09^{n}	57.57±1.22^{a}	nd	65.97±1.40^{a}	**127.93±2.71^{a}**	0.67±0.01^{e}	10.89±0.23^{gh}	0.92±0.02^{ef}	nd	nd	0.94±0.02^{kl}	nd	5.05±0.11^{bc}	nd	nd	nd	nd	nd	nd	**47.28±1.00^{f}**
OC%100	6.11±0.13l^{m}	nd	nd	8.88±0.19^{j}	**15.10±0.32^{r}**	1.30±0.03^{b}	15.71±0.33^{e}	0.66±0.01^{fg}	nd	0.43±0.01^{fg}	1.99±0.04^{bc}	0.34±0.01^{e}	8.60±0.18^{a}	nd	nd	nd	nd	nd	nd	**54.05±1.15^{e}**

Table 3. continue

Sample	F1	F2	F3	Total F	A1	A2	A3	A4	A5	A6	A7	Total A	Procyanidins	DP	Total P	C1	C2	C3	C4	Total C
**RJ-PC**	4.64±0.10^{de}	nd	nd	**4.64±0.10^{d}**	2.01±0.04^{b}	12.83±0.27^{d}	5.60±0.12^{cd}	nd	1.25±0.03^{de}	nd	nd	**21.70±0.46^{d}**	**12.08±0.26^{f}**	9.50	**190.77 ^{d}**	nd	nd	nd	nd	**nd**
**AJ-PC**	nd	nd	nd	**nd**	1.80±0.04^{c}	nd	5.34±0.11^{d}	12.56±0.27^{b}	1.11±0.02^{de}	nd	nd	**20.81±0.44^{d}**	**9.42±0.20^{g}**	6.46	**150.96 ^{e}**	0.03±0.00^{a}	21.93±0.47^{a}	0.27±0.01^{a}	nd	**22.22±0.47^{a}**
**PJ-PC**	nd	nd	nd	**nd**	1.21±0.03^{d}	nd	4.76±0.10^{e}	10.77±0.23^{c}	0.85±0.02^{e}	nd	nd	**17.59±0.37^{e}**	**4.26±0.09^{l}**	6.31	**135.56 ^{f}**	nd	nd	0.10±0.00^{b}	0.78±0.02^{e}	**0.88±0.02^{def}**
**SJ-PC**	4.28±0.09^{e}	nd	nd	**4.28±0.09^{d}**	1.82±0.04^{c}	nd	6.04±0.13^{c}	13.21±0.28^{b}	8.43±0.18^{c}	nd	nd	**29.51±1.63^{c}**	**16.75±0.36^{d}**	nd	**177.64 ^{d}**	nd	nd	nd	0.98±0.02^{d}	**0.98±0.02^{de}**
**SCJ-PC**	8.48±0.18^{a}	30.31±0.64^{a}	nd	**38.79±0.82^{c}**	1.69±0.04^{c}	21.98±0.47^{b}	6.01±0.13^{c}	13.22±0.28^{b}	1.04±0.02^{e}	7.42±0.16^{b}	nd	**51.35±3.09^{a}**	**25.56±0.54^{a}**	1.59	**173.29 ^{d}**	nd	nd	nd	0.63±0.01^{fg}	**0.63±0.01^{efg}**
**PC%100**	nd	nd	nd	**nd**	2.65±0.06^{a}	nd	8.30±0.18^{b}	17.30±0.37^{a}	1.62±0.03^{d}	nd	nd	**29.87±0.63^{c}**	**4.49±0.10^{l}**	4.01	**278.06 ^{b}**	nd	nd	nd	2.14±0.05^{a}	**2.14±0.05^{b}**
**RJ-WC**	4.39±0.09^{de}	nd	nd	**4.39±0.09^{d}**	nd	13.82±0.29^{cd}	nd	nd	nd	1.39±0.03^{c}	2.06±0.04^{a}	**17.27±0.37^{e}**	**11.56±0.25^{f}**	3.54	**108.28 ^{h}**	nd	nd	nd	nd	**nd**
**AJ-WC**	nd	nd	nd	**nd**	nd	nd	nd	nd	nd	nd	nd	**nd**	**8.18±0.17^{gh}**	4.47	**71.14 ^{k}**	nd	nd	nd	nd	**nd**
**PJ-WC**	nd	nd	nd	**nd**	nd	nd	nd	nd	nd	nd	nd	**nd**	**5.12±0.11^{k}**	4.73	**50.66 ^{n}**	nd	nd	nd	nd	**nd**
**SJ-WC**	3.54±0.08^{f}	nd	nd	**3.54±0.08^{d}**	nd	nd	nd	nd	9.11±0.19^{b}	nd	nd	**9.11±0.19^{f}**	**15.77±0.33^{d}**	nd	**82.42 ^{i}**	nd	nd	nd	nd	**nd**
**SCJ-WC**	8.38±0.	31.49±	43.21±	**83.08±1.**	nd	23.31	9.34±0.2	nd	nd	nd	nd	**32.65±0.**	**22.62±0.48^{b}**	1.60		nd	nd	nd	nd	**nd**

	18^{a}	0.67^{a}	0.92^{a}	**76^{a}**		±0.49^{ab}	0^{a}					**69^{b}**			**238.18 ^{c}**					
**WC%100**	nd	nd	nd	**nd**	nd	nd	nd	nd	nd	nd	nd	**nd**	**3.83±0.08^{m}**	nd	**54.30 ^{m}**	nd	nd	nd	nd	**nd**
**RJ-YC**	4.81±0.10^{d}	nd	nd	**4.81±0.10^{d}**	nd	13.37 ±0.28^{cd}	nd	nd	nd	1.44±0.03^{c}	1.96±0.04^{a}	**16.77±0.36^{e}**	**14.28±0.30^{e}**	4.39	**128.50 ^{f}**	nd	nd	nd	0.45±0.01^{h}	**0.45±0.01^{fg}**
**AJ-YC**	nd	nd	nd	**nd**	nd	nd	nd	nd	nd	nd	nd	**nd**	**8.13±0.17^{gh}**	5.09	**77.70 ^{j}**	nd	nd	nd	0.24±0.01^{k}	**0.24±0.01^{g}**
**PJ-YC**	nd	nd	nd	**nd**	nd	nd	nd	nd	nd	nd	nd	**nd**	**5.50±0.12^{jk}**	4.98	**60.18 ^{l}**	nd	nd	nd	0.29±0.01^{jk}	**0.29±0.01^{g}**
**SJ-YC**	3.30±0.07^{f}	nd	nd	**3.30±0.07^{d}**	nd	nd	nd	nd	8.85±0.19^{bc}	nd	nd	**8.85±0.19^{f}**	**15.66±0.33^{d}**	nd	**115.95 ^{g}**	nd	nd	nd	0.59±0.01^{g}	**0.59±0.01^{efg}**
**SCJ-YC**	8.41±0.18^{a}	30.73±0.65^{a}	42.52±0.90^{a}	**81.65±1.73^{a}**	nd	22.56 ±0.48^{b}	nd	nd	nd	8.30±0.18^{a}	nd	**30.86±0.65^{bc}**	**23.08±0.49^{b}**	1.68	**279.20 ^{b}**	nd	nd	nd	0.51±0.01^{h}	**0.51±0.01^{efg}**
**YC%100**	nd	nd	nd	**nd**	nd	nd	nd	nd	nd	nd	nd	**nd**	**2.59±0.05^{n}**	nd	**62.19 ^{l}**	nd	nd	nd	1.31±0.03^{c}	**1.31±0.03^{cd}**
**RJ-OC**	5.42±0.12^{c}	nd	nd	**5.42±0.12^{d}**	nd	14.72 ±0.31^{c}	nd	nd	nd	1.47±0.03^{c}	2.08±0.04^{a}	**18.27±0.39^{e}**	**9.38±0.20^{g}**	3.06	**117.99 ^{g}**	nd	nd	0.06±0.00^{c}	0.51±0.01^{h}	**0.57±0.01^{efg}**
**AJ-OC**	nd	nd	nd	**nd**	nd	nd	nd	nd	nd	nd	nd	**nd**	**7.86±0.17^{h}**	4.74	**85.05 ^{i}**	nd	nd	nd	0.33±0.01^{j}	**0.33±0.01^{g}**
**PJ-OC**	nd	nd	nd	**nd**	nd	nd	nd	nd	nd	nd	nd	**nd**	**5.71±0.12^{j}**	4.33	**60.39 ^{l}**	nd	nd	nd	0.37±0.01^{j}	**0.37±0.01^{g}**
**SJ-OC**	4.16±0.09^{e}	nd	nd	**4.16±0.09^{d}**	nd	nd	nd	nd	9.79±0.21^{a}	nd	nd	**9.79±0.21^{f}**	**16.34±0.35^{d}**	nd	**105.66 ^{h}**	nd	nd	nd	0.66±0.01^{fg}	**0.66±0.01^{efg}**
**SCJ-OC**	7.61±0.16^{b}	27.40±0.58^{b}	41.86±0.89^{a}	**76.87±1.63^{b}**	nd	24.23 ±0.51^{a}	nd	nd	nd	8.48±0.18^{a}	nd	**32.70±0.69^{b}**	**20.01±0.42^{c}**	1.50	**304.79 ^{a}**	nd	nd	nd	0.70±0.01^{ef}	**0.70±0.01^{efg}**
**OC%100**	nd	nd	nd	**nd**	nd	nd	nd	nd	nd	nd	nd	**nd**	**2.68±0.06^{n}**	nd	**71.83 ^{k}**	nd	nd	nd	1.65±0.04^{b}	**1.65±0.04^{c}**

Polyphenols. and carotenoids-mg/100 ml; values (mean of three replications) followed by the same letter within the same column were not significantly different (p ≤0.05) according to Tukey's test. FL3 – flavan-3-ols; Fl3 1 – procyanidin B2; Fl3 2 – procyanidin B4; Fl3 3 – (-)-epicatechin; PA – phenolic a[illegible] chlorogenic acid; PA 2 - 5-O-caffeoylquinic acid; PA 3 - 4-O-caffeoylquinic acid; PA 4 - ferulic acid-hexoside; PA 5 - 3-O-feruloylquinic acid; PA 6 - 4-O-feruloylquinic acid; PA 7 - caffeic acid –hexoside; PA 8 - di-ferulic acid derivative; PA 9 - dicaffeoylquinic acid; PA 10 - cis-5-p-coumaroylquinic acid; PA 11 – [illegible] acid; PA 12 - 3-O-caffeoylquinic acid; PA 13 - ferulic acid di-hexoside; F – flavonols, F 1 - quercetin-3-galactoside; F 2 – genistin; A - anthocyanins; A2 - cyanidin-3-O-xylosyl-glucosylgalactoside; A1 - cyanidin-3-O-xylosyl-galactoside; A3 - cyanidin-3-O-xylosyl-cinpoyl-glucosylgalactoside; A4 - cyanidin-3[illegible] feruloyl-glucosylgalactoside; A5 - cyanidin-3-O-xylosyl-p-coumaroylglucosyl-galactoside; A6 - cyanidin-3-O-glucosyl-rutinoside; A7 - cyanidin-3-arabinoside; TP – total polyphenolic compounds; C1 - α-cryptoxanthin (zeinoxanthin); C2 – β-carotene; C3 - pheophytin a; C4 – lutein; TC – totacl carotenoi[illegible] detected.
